# Coinhibition of the deubiquitinating enzymes, USP14 and UCHL5, with VLX1570 is lethal to ibrutinib- or bortezomib-resistant Waldenstrom macroglobulinemia tumor cells

**DOI:** 10.1038/bcj.2016.93

**Published:** 2016-11-04

**Authors:** A Paulus, S Akhtar, T R Caulfield, K Samuel, H Yousaf, Y Bashir, S M Paulus, D Tran, R Hudec, D Cogen, J Jiang, B Edenfield, A Novak, S M Ansell, T Witzig, P Martin, M Coleman, V Roy, S Ailawadhi, K Chitta, S Linder, A Chanan-Khan

**Affiliations:** 1 Department of Cancer Biology, Mayo Clinic, Jacksonville, FL, USA; 2 Division of Hematology and Oncology, Mayo Clinic, Jacksonville, FL, USA; 3 Department of Molecular Neuroscience, Mayo Clinic, Jacksonville, FL, USA; 4 Department of Laboratory Medicine and Pathology, Mayo Clinic, Jacksonville, FL, USA; 5 Department of Hematology and Internal Medicine, Mayo Clinic, Rochester, MN, USA; 6 Department of Medicine, Weill Cornell Medical College, Cornell, NY, USA; 7 Institute for Oncology-Pathology, Cancer Center Karolinska, Karolinska Institute, Stockholm, Sweden

## Abstract

The survival of Waldenstrom macroglobulinemia (WM) tumor cells hinges on aberrant B-cell receptor (BCR) and MYD88 signaling. WM cells upregulate the proteasome function to sustain the BCR-driven growth while maintaining homeostasis. Clinically, two treatment strategies are used to disrupt these complementary yet mutually exclusive WM survival pathways via ibrutinib (targets BTK/MYD88 node) and bortezomib (targets 20 S proteasome). Despite the success of both agents, WM patients eventually become refractory to treatment, highlighting the adaptive plasticity of WM cells and underscoring the need for development of new therapeutics. Here we provide a comprehensive preclinical report on the anti-WM activity of VLX1570, a novel small-molecule inhibitor of the deubiquitinating enzymes (DUBs), ubiquitin-specific protease 14 (USP14) and ubiquitin carboxyl-terminal hydrolase isozyme L5 (UCHL5). Both DUBs reside in the 19 S proteasome cap and their inhibition by VLX1570 results in rapid and tumor-specific apoptosis in bortezomib- or ibrutinib-resistant WM cells. Notably, treatment of WM cells with VLX1570 downregulated BCR-associated elements BTK, MYD88, NFATC, NF-κB and CXCR4, the latter whose dysregulated function is linked to ibrutinib resistance. VLX1570 administered to WM-xenografted mice resulted in decreased tumor burden and prolonged survival (*P*=0.0008) compared with vehicle-treated mice. Overall, our report demonstrates significant value in targeting USP14/UCHL5 with VLX1570 in drug-resistant WM and carries a high potential for clinical translation.

## Introduction

Waldenstrom macroglobulinemia (WM) is an indolent yet incurable B-cell lymphoma, in which aberrant B-cell receptor (BCR), Toll-like receptor and MYD88 signaling cooperatively drive tumor cell survival. This observation has been clinically exploited with the BTK inhibitor, ibrutinib. Although capable of inducing remission in ~91% of WM patients, the majority of patients on ibrutinib therapy exhibit partial response, with no complete remissions observed.^1^ Moreover, the phenomenon of ibrutinib resistance is increasingly being reported in other B-cell cancers,^2, 3, 4, 5, 6, 7^ and although WM patients demonstrate durable responses to ibrutinib, it is anticipated that when these patients relapse (or become refractory to the drug), their survival outcome will be poor.^8, 9^ These observations underscore the plasticity of WM cells to adapt to targeted therapeutic stress through modulation of other oncogenic survival mechanisms, signifying a need for continued development of new treatment strategies.

To sustain BCR/Toll-like receptor-driven growth and maintain optimal cellular homeostasis, WM cells upregulate proteasome function.^10, 11^ Clinical benefit derived through proteasome inhibitor (PI) therapy has been clearly established in several B-cell cancers, including WM.^12^ PI-based management of disease yields responses in ~85% of relapsed/refractory WM patients.^13, 14, 15, 16^ Current PI (bortezomib, carfilzomib and ixazomib) are designed to preferentially inhibit the β-subunit catalytic activity of the 20 S proteasome core. However, the 26 S proteasome contains several distinct components (with exclusive functions), each whose roles in supporting tumorigenesis remain underexplored and for which no therapeutic agents are currently approved.

The 19 S regulatory particle of the mammalian proteasome contains deubiquitinating enzymes (DUBs) that unfold and remove ubiquitin moieties from proteins before their entry into the 20 S β-catalytic core.^17^ Two of these, ubiquitin-specific protease 14 (USP14) and ubiquitin carboxyl-terminal hydrolase isozyme L5 (UCHL5), reversibly associate with the proteasome,^18^ and, collectively, their dysregulated activity has been linked to tumor cell survival,^19^ metastasis^20^ and poor clinical outcome.^21, 22^ We have recently described the effects of a chemical inhibitor (b-AP15), which preferentially targets USP14 and UCHL5 and induces robust tumor cell death in drug-resistant lymphoplasmacytic cells *in vitro*.^23^ Herein, we extended our investigations to define the *in vitro* and *in vivo* antineoplastic activity of a clinical grade DUB inhibitor, VLX1570, notably in WM cells that are resistant to β5-targeting PI or BTK inhibition. Compared with b-AP15, VLX1570 displays enhanced solubility, stability and target residence time and is currently being investigated in relapsed/refractory multiple myeloma patients.

## Materials and methods

### Primary WM cells, cell lines, cell culture and reagents

WM cell lines and derived bortezomib-resistant (BR) suclones,^24, 25^ as well as ibrutinib-resistant (IR) subclones,^26, 27^ were used in experiments, as reported previously.^23, 25^ Primary WM patient cells (CD19+/CD138+ sorted) were obtained from the Predolin Biobank (Mayo Clinic, Rochester MN, USA) after approval by the Mayo Clinic Institutional Review Board. Heparinized peripheral blood from healthy human donors was obtained and peripheral blood mononuclear cells (PBMCs) were extracted, as described previously.^28^ All cells were cultured according to conditions previously described by us.^23^ VLX1570 and b-AP15 were provided as gifts from Vivolux AB (Uppsala, Sweden). RPMI, penicillin, streptomycin, tetramethylrhodamine, methyl ester (TMRM) and fetal bovine serum were purchased from Life Technologies (Carlsbad, CA, USA). Ibrutinib and bortezomib were purchased from Sellekhem (Houston, TX, USA). Annexin-V/Propidium Iodide Apoptosis Staining Kit was purchased from BD Biosciences (San Jose, CA, USA).

### Cell death, proliferation and apoptosis assays

MTS assay was used to determine the half-maximal inhibitory concentration values and proliferation rate/viability; apoptosis was determined using the Annexin-V/Propidium Iodide Binding Assay Kit from BD Biosciences (San Diego, CA, USA) according to the manufacturer's instructions and previously described methods.^23, 25^

### Determination of MOMP

Cells were treated with VLX1570 for 12 h and assessed for mitochondrial outer membrane permeability (MOMP) using TMRM (Life Technologies) in a manner similar to that reported by us previously.^23, 25^

### CXCR4 surface receptor analysis

For staining of CXCR4 on WM tumor cells, cells were washed two times with cold phosphate-buffered saline and suspended in 300 μl of binding buffer (phosphate-buffered saline solution with 2% fetal bovine serum). Cell were divided in three tubes: unstained, isotype control and those with antibody against CD184/CXCR4 (BioLegend, San Diego, CA, USA). Five microliters of antibody was added and cells were incubated for 30 min at room temperature. Tumor cells were washed two times with cold phosphate-buffered saline and suspended in 100 μl of 4% paraformaldehyde in phosphate-buffered saline solution (Affymetrix Inc., Santa Clara, CA, USA; 19943 1LT) followed by an analysis using a BD Accuri C6 Flow Cytometer (Franklin Lakes, NJ, USA). FCS Express 4 (*De novo* Softwares, Los Angeles, CA, USA) was used to analyze the data.

### HA-Ub-VS labeling of USP14 and UCHL5

WM cells were treated with dimethyl sulfoxide (DMSO) or VLX1570 (250 nm) for 3 h, harvested and lysed in RIPA lysis buffer followed by centrifugation at 13 000  r.p.m. for 5 min. Total protein (20 μg) was labeled with 5 μm HA-tagged ubiquitin-vinyl sulfone (HA-Ub-VS; Boston Biochem, Cambridge, MA, USA) probe for 1, 10, 20 or 30 min at 37 °C and then subjected to western blotting with anti-USP14 or anti-UCHL5 antibodies.

### Real-time quantitative PCR

RNA was isolated from the cells using miRCURY Exiqon (Exiqon, Woburn, MA, USA, 300110) and was later quantified using Thermo NanoDrop 2000c (ThermoFisher Scientific, Waltham, MA, USA). cDNA was prepared with RNA at the concentration of 1 μg with cDNA High Capacity Reverse Transcription Kit (ThermoFisher Scientific, 4368813). Samples were diluted to 2 ng for real-time reaction using LightCycler 96 system (Roche Diagnostics, Mannheim, Germany). Taq-Man probes: BCL-xL(Hs00236329) BTK(Hs00975865_m1), CXCR4(Hs00607978_s1), MYD88 (Hs01573837_g1) and NFATC2(hs00905451_m1) were obtained from Life Technologies.

### Human WM xenograft model

Animal experiments were performed with the approval of the Institutional Animal Care and Use Committee of Mayo Clinic. A xenograft model of WM was established as described previously.^27^

### Calculation of drug combination effects

The pharmacobiological interaction between VLX1570 and ibrutinib was examined in WM cells based on the Chou-Talalay principle^29^ using the CompuSyn software program (ComboSyn Inc., Paramus, NJ, USA). WM cells were exposed to different concentrations of VLX1570 and ibrutinib for 24 h in quadruplicate and viability was examined using the CellTiter Glo assay (Promega Corp., Madison, WI, USA), according to the manufacturer's instructions. Cell viability data were expressed as the fraction of antiproliferative activity (Fraction affected, Fa) by the individual drugs or the combination in drug-treated cells from which a combination index (CI) value was derived. An additive effect is indicated by a CI of 1, an antagonistic effect is denoted by CI >1 and a synergistic effect is indicated by a CI <1.

### Statistical analysis

Statistical evaluation was performed using GraphPad Prism 6 (GraphPad Software Inc., La Jolla, CA, USA). Standard error bars were derived from the standard deviation of values from average values from three or more experiments. *P*-values were calculated using a Student's *t*-test of two data array sets in triplicates with one-tailed and two-sample unequal variance. The Kaplan–Meier method was used to visualize overall survival differences between VLX1570-treated mice and mice given vehicle only. A log-rank statistic was used to establish the statistical significance of the difference between survival distributions.

Please see the Supplementary Materials and methods for additional information.

## Results

### USP14 and UCHL5 is highly expressed in WM cells

**Figure 1:**
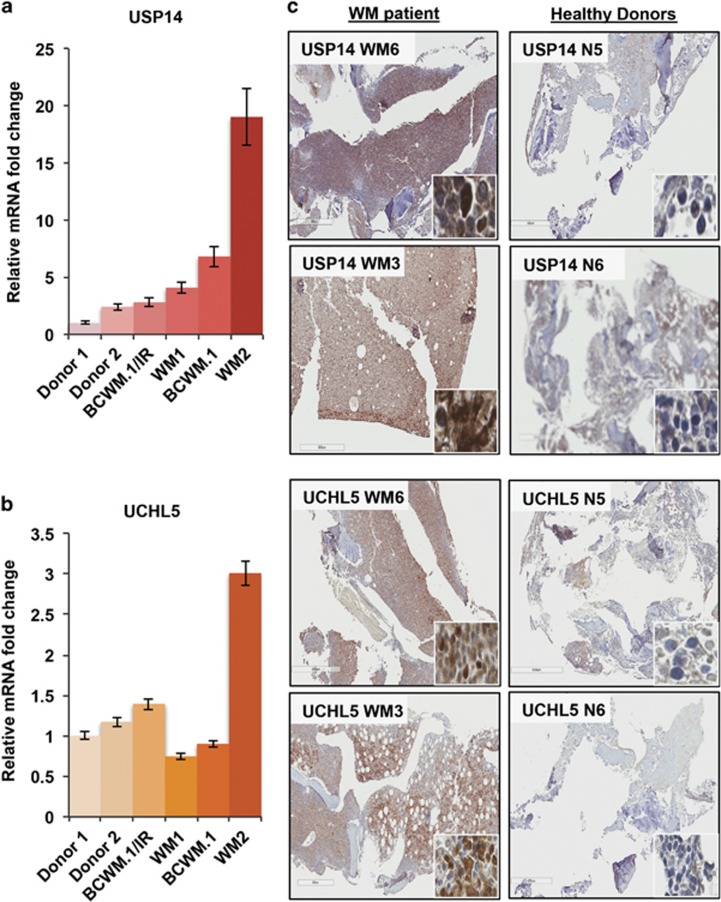
USP14 and UCHL5 expression in primary WM cells, WM cell lines and bone marrow aspirate from WM patients. USP14/UCHL5 mRNA expression was examined in PBMCs from healthy donors (donors 1 and 2), primary WM cells (WM1 and WM2) the BCWM.1 cell line and its IR BCWM.1/IR derivative by real-time quantitative PCR (qPCR). POLR2A was used as a reference gene and fold change was calculated relative to mRNA expression in donors. (**a**) USP14 mRNA was increased in all WM cells tested with highest expression observed in WM2 patient cells (19-fold increase). (**b**) Similarly, UCHL5 mRNA was assessed and demonstrated more variability, with highest expression (~3-fold increase) in WM2 patient cells. (**c**) Immunohistochemistry (IHC) analysis was conducted on bone marrow aspirate from WM patients (total *n*=6, WM6 and WM3 shown as representatives) and stained for USP14 and UCHL5. Expression, distribution and staining intensity were noted to be greater in bone marrow of WM patients compared with that of healthy donors (N5 and N6). Inset IHC images are shown at × 40 magnification.

USP14 and UCHL5 are known to be expressed in a variety of cell types including hematopoietic cells.^17, 30^ We first quantified their expression in WM cell lines as well as in CD19+/CD138+-sorted primary WM tumor cells. Compared with PBMCs from healthy donors, USP14 mRNA expression was significantly elevated in WM tumor cell lines and primary WM cells (5.4-fold median increase; range, 2.8–19-fold) (Figure 1a). For UCHL5, we found variable expression, where compared with healthy donors, UCHL5 mRNA was higher only in IR WM cells (BCWM.1/IR, 1.4-fold increase) and primary cells from a WM patient with relapsed/refractory disease (WM2, 3-fold increase) (Figure 1b). These findings are largely consistent with our previous report on USP14/UCHL5 protein expression in BR WM cell lines.^23^ Immunostaining of bone marrow biopsies from patients with a confirmed diagnosis of WM (Figure 1c, WM6 and WM3) demonstrated increased staining intensity and overall distribution of both USP14 and UCHL5 compared with marrow from normal healthy donors (Figure 1c, N5 and N6). Altogether, these findings affirmed that both proteasome-associated DUBs are upregulated and their expression is increased in human WM cells.

### VLX1570 binds and inhibits the activity of USP14 and UCHL5

**Figure 2:**
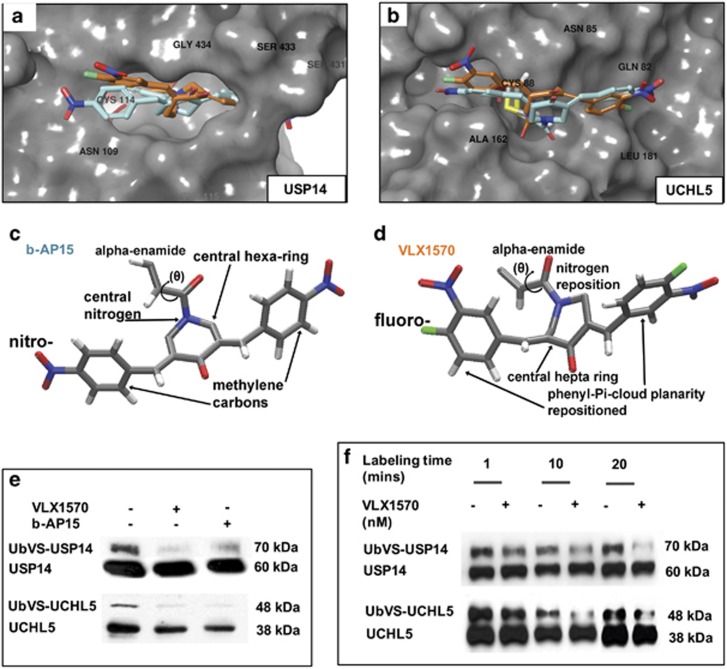
Comparative analysis of b-AP15 and VLX1570 binding with USP14 or UCHL5. The chemical structures of VLX1570 (orange) and b-AP15 (turquoise) were modeled and docked to USP14 and UCHL5 *in silico*. (**a**) Overlay shows how b-AP15 and VLX1570 interface with USP14 residues that are critical for binding affinity, notably for the covalent docked linkage at the –SH thiol from Cys114. (**b**) Similarly, overlay of b-AP15 and VLX1570 in UCHL5 shows their relative alignment and optimal positioning to covalently dock at the –SH thiol from Cys88. Three-dimensional (3D) chemical renderings of (**c**) b-AP15 and (**d**) VLX1570 reveal key structural differences that result in enhanced activity of VLX1570. (**e**) A competitive enzyme assay using the HA-Ub-VS DUB labeling probe was carried out in BCWM.1 cells to identify whether the DUB inhibitors reduced labeling of USP14/UCHL5 in live WM cells. BCWM.1 cells were treated with DMSO, b-AP15 or VLX1570 for 3 h and thereafter incubated with the HA-Ub-VS probe for 30 min. After immunoblotting with USP14 or UCHL5 antibodies, we observed the fraction of labeled USP14 or UCHL5 was notably reduced in DUB inhibitor-treated vs DMSO-treated cells. (**f**) In a similar manner, we labeled DMSO or VLX1570-treated BCWM.1 cells with HA-Ub-VS for 1, 10 and 20 min to determine if reduction in DUB activity occurred at an earlier stage. We noted that labeled USP14 and UCHL5 were markedly decreased between 10 and 15 min after suicide-substrate labeling.

We have recently reported on the lead optimization of b-AP15, from which VLX1570 emerged as the most promising analog, suitable for clinical use.^31^ We modeled VLX1570 and b-AP15 side by side and performed a comparative *in silico* docking analysis on their binding capabilities with USP14 and UCHL5 (Figures 2a and b; b-AP15 in turquoise and VLX1570 in orange). VLX1570 preferentially binds the Cys88 residue of UCHL5 and interfaces with a thiol of USP14 at residue Cys114 via a 1,4-Michael's addition reaction, potentially forming a covalent bond. These binding interactions are nearly identical to those of b-AP15; however, notable differences between the compounds account for superiority of VLX1570 over b-AP15. b-AP15 has two nitro moieties (one per phenyl ring), whereas VLX1570 has nitro moieties and also carries a fluoro on each phenyl at the *para*-position for a connection with a methylene carbon. b-AP15 has a central nitrogen-substituted hexyl moiety, whereas VLX1570 has a nitrogen-substituted heptyl moiety (compare Figures 2c and d). Improved affinity for its targets is most likely due to the electrostatics from the two fluoro atoms. Additionally, although both compounds have an α-enamide at the warhead position, the nitrogen-substituted alkane ring is slightly modified for VLX1570 so that a better α-enamide position is available for approaching the Cys residue in the active site of the target proteins. The precovalent docked poses from VLX1570 are superior to the precovalent docked poses of b-AP15, indicating that the initial approach of the VLX1570 is superior for forming optimal geometry of orientation required for the thiol addition reaction. In general, VLX1570 is better able to form covalent docking owing to better position before the reaction. Thus, these chemical differences predict for VLX1570 to be significantly more stable and soluble with improved *in vivo* clearance.

Next, to determine the binding capacity of VLX1570 in a cellular system, we used an enzyme-based competition assay, which uses a DUB suicide-substrate probe (HA-UB-VS) that competes with active DUBs for binding to other molecules. We observed at 30 min that VLX1570 inhibited the activity of both USP14 and UCHL5 in a manner similar to b-AP15, as noted by a decrease in UbVS-labeled DUBs (Figure 2e). To determine if this effect occurred at an earlier time point, we probed for labeled and unlabeled USP14 and UCHL5 and observed a time-dependent decrease in labeled fractions of both DUBs where UbVS-tagged USP14 or UbVS-tagged UCHL5 were indeed reduced as early as 10 min after labeling (Figure 2f).

### Pharmacologic inhibition of USP14 and UCHL5 with VLX1570 induces apoptosis in drug-resistant WM cells and is associated with accumulation of high-molecular-weight polyubiquitinated protein conjugates

**Figure 3:**
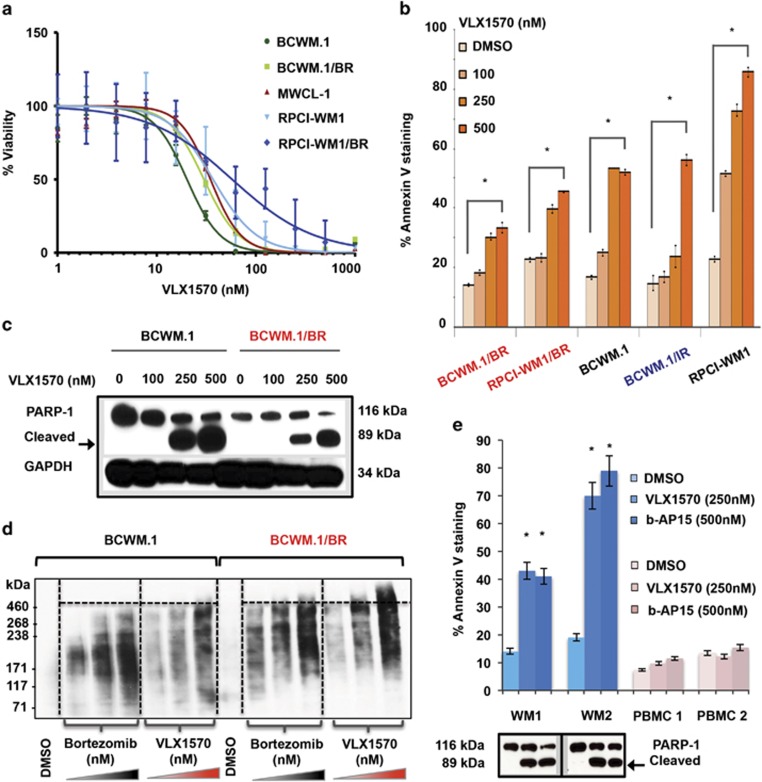
VLX1570 induces loss of cell viability, apoptosis and accumulation of high-molecular-weight protein conjugates in drug-resistant WM cells. (**a**) Seventy-two hours MTS assay was conducted to assess sensitivity of WM cell lines (BCWM.1, MWCL-1 and RPCI-WM1) including their BR derivatives (BCWM.1/BR and RPCI-WM1/BR) towards VLX1570. Median EC_50_ (half-maximal effective concentration) of the cell lines tested was 29.96 nm (range, 20.2–93.59 nm). EC_50_ values for each individual cell lines are presented in Supplementary Table 1. (**b**) WM cell lines, including BR or IR subclones, were treated with DMSO or VLX1570 at indicated concentrations for 12 h and stained with Annexin-V and propidium iodide followed by flow cytometry to examine apoptosis. VLX1570 induced significant apoptosis (denoted by *) in all WM cell lines tested in a dose-dependent manner with (*P*=0.01–0.00003). The comparative percentage (and statistical significance) of % Annexin-V positivity in VLX1570-treated cell lines is presented in Supplementary Table 2. (**c**) Immunoblotting for poly (ADP-ribose) polymerase-1 (PARP-1) cleavage confirmed execution of apoptosis (representative BCWM.1 and its BR derivative, BCWM.1/BR shown). (**d**) Polyubiquitinated protein accumulation was examined in BCWM.1 and BCWM.1/BR cells after treatment with DMSO, bortezomib or VLX1570 at indicated concentrations for 3 h followed by isolation of protein lysates and immunoblotting with anti-polyubiquitin antibody. Notably, VLX1570 treatment in BCWM.1/BR cells resulted in higher-molecular-weight ubiquitinated protein buildup compared with bortezomib treatment. (**e**) Apoptosis in CD19+/CD138+ primary WM cells from two patients (WM1 and WM2) as well as PBMCs from healthy donors (PBMC 1 and PBMC 2) treated with either b-AP15 or VLX1570 at indicated concentrations for 12 h was examined as in (**b**). VLX1570 and b-AP15 induced nearly equivalent levels of significant apoptotic cell death in primary WM cells, whereas insignificant apoptosis was noted in PBMCs (relative to DMSO-treated cells). Apoptotic cell death in WM1 and WM2 cells was confirmed by immunoblotting for PARP-1 cleavage.

We profiled the sensitivity of established WM cell lines^32^ as well as their drug-resistant subclones towards VLX1570 by 72 h MTS assay and noted all WM cells to be sensitive to treatment. BCWM.1 cells were most sensitive, undergoing ~50% loss of viability in the presence of ~20 nm of VLX1570 (EC_50_ 20.22 nm). Notably, cytotoxic activity of VLX1570 was maintained in its BCWM.1/BR cells, which exhibited an EC_50_ of 29 nm (Figure 3a and Supplementary Table 1).

Next, we investigated whether VLX1570-mediated loss of viability in WM cells was due to apoptotic cell death. Compared with DMSO-treated cells, VLX1570 caused significant (*P<*0.01–0.00003) apoptosis by 12 h in a dose-dependent manner in all WM cell lines tested, including IR and BR subclones (Figure 3b, Supplementary Figure 1 and Supplementary Table 2). Apoptosis was secondarily confirmed by PARP-1 cleavage (Figure 3c). Moreover, in BR cells, VLX1570 treatment was associated with the accumulation of higher-molecular-weight polyubiquitinated protein conjugates (~460 kDa) when compared with equivalent concentrations of bortezomib treatment in the same cells (Figure 3d).

We also treated primary WM tumor cells with VLX1570 and noted apoptosis and PARP-1 cleavage at concentrations as low as 250 nm (vs b-AP15 500 nm) (Figure 3e, Supplementary Figure 2 and Supplementary Table 3). Contrastingly, exposure of PBMCs from healthy donors to VLX1570 or b-AP15 resulted in negligible cell death. These data indicate that VLX1570 is minimally toxic to non-tumor cells from the peripheral blood of healthy donors but induces significant cell death in malignant WM cells, irrespective of their resistance to β5-targeting PI bortezomib or ibrutinib.

### VLX1570 modulates ER stress machinery and perturbs mitochondrial transmembrane integrity leading to activation of the intrinsic apoptotic cascade

**Figure 4:**
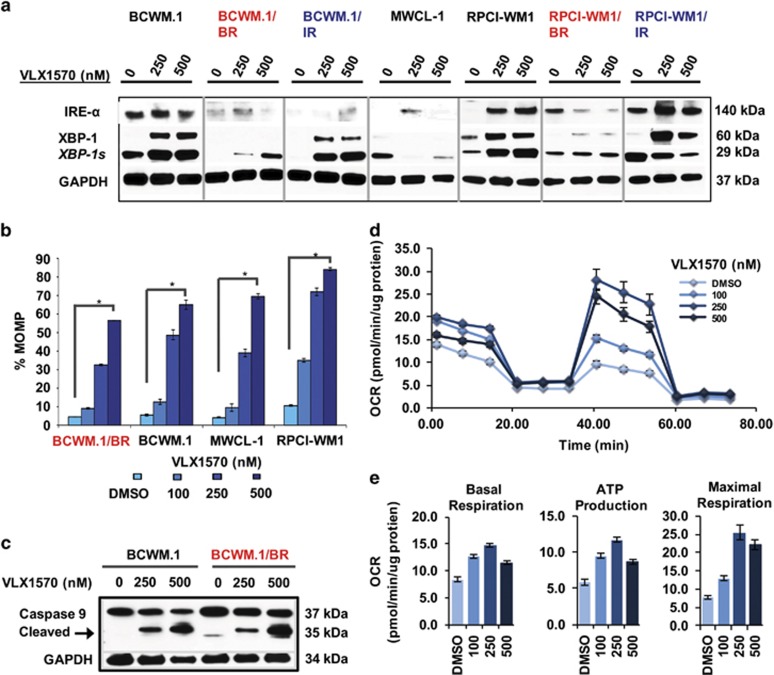
VLX1570 invokes ER stress machinery and mitochondrial damage in WM cells. (**a**) WM cell lines including BR (red font) and IR subclones (blue font) were treated with VLX1570 at indicated concentrations for 12 h and subjected to immunoblotting with anti-IREα, anti-XBP-1, XPB-1 s and GAPDH antibodies. ER stress-associated protein XBP-1 (unspliced) as well as XBP1s (spliced) were markedly induced by VLX1570. (**b**) MOMP was measured in relation to TMRM fluorescence in all WM cell lines and TMRM-negative cells were calculated to represent (%) MOMP (four representative cell lines shown). MOMP was significantly induced in VLX1570-treated WM cells in a dose-dependent manner (denoted by *P=0.0000001–0.00025). (**c**) Immunoblot analysis demonstrated cleavage of caspase-9 after VLX1570 treatment (representative BCWM.1 and BCWM.1/BR shown). (**d**) Mitochondrial bioenergetics were measured at baseline in BCWM.1 cells followed by inhibition of the mitochondrial ATP production (introduction of oligomycin), uncoupling oxidative phosphorylation (OP) and ATP production causing maximal mitochondrial respiration (introduction of FCCP) and finally complete inhibition of the OP by combination of rotenone and antimycin A. Oxygen consumption rate (OCR) is displayed in values normalized to total protein content. VLX1570-treated cells (100, 250 and 500 nm) were analyzed side by side with DMSO-treated control cells. (**e**) Treatment of cells with VLX1570 caused concentration-dependent biphasic changes of all analyzed parameters: basal respiration (left), ATP production (middle) and maximal respiration (right); displaying maximal values in 250 nm concentration.

USP4/UCHL5 inhibition has been shown to have a prominent effect on ER and cell stress proteins.^33, 34^ Therefore, we looked at ER stress-associated proteins, and in line with prior observations,^30, 34^ we noted ER stress machinery such as XBP-1 (and its spliced active form, XBP-1 s), which are primary effectors of ER stress induction,^35^ to be upregulated by VLX1570 in WM cell lines (Figure 4a). IRE-1α levels did not show any consistent pattern of alteration following VLX1570 treatment. These differential changes (in IRE-1α) may reflect underlying differences in the degree of basal ER stress present across the different WM cell lines and thus their response to VLX1570.

Disruption of the mitochondrial transmembrane potential (Δψ_m_), through an increase in MOMP, can occur in response to a strong (but ultimately futile) ER stress response and signifies engagement of the intrinsic apoptotic cascade.^36, 37^ We found MOMP to be significantly induced in WM cell lines exposed to VLX1570 (*P<*0.00025–0.000001), as noted by % decrease of TMRM fluorescence in drug-treated cells relative to their untreated counterparts (Figure 4b, Supplementary Figure 3 and Supplementary Table 4). Activation of the intrinsic apoptotic pathway was also verified by cleavage of caspase-9 (Figure 4c).

To further understand the mitochondrial bioenergetics preceding these events, we measured mitochondrial oxygen consumption rate and noted that mitochondrial respiration is triggered as early as 2 h after VLX1570 treatment (Figure 4d). Increasing concentrations of VLX1570 caused elevation of basal respiration and ATP production, as well as uncoupler induced maximal respiration (Figure 4e). Maximal mitochondrial respiratory activity was reached by VLX1570 250 nm and higher concentrations resulted in collapse of all monitored parameters, likely due to mitochondrial exhaustion, ultimately leading to mitochondria-mediated apoptosis.

### VLX1570 downregulates BCR-signalosome components and their end effectors, as well as CXCR4 expression

**Figure 5:**
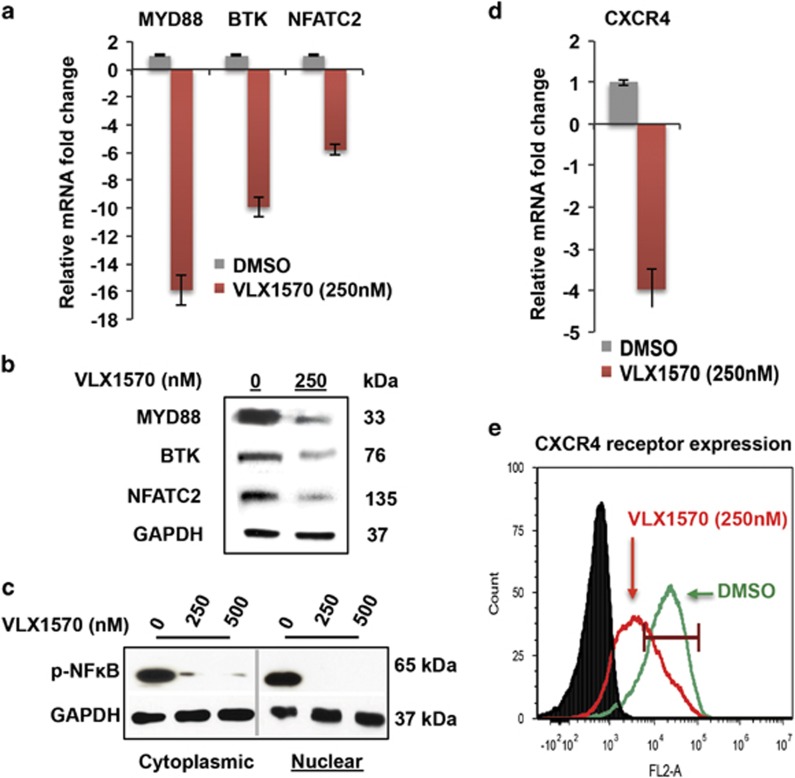
Effects of VLX1570 on BCR-signalosome components and CXCR4 expression in WM cells. (**a**) BCWM.1/IR cells were treated with DMSO or VLX1570 (250 nm) for 3 h and mRNA expression of BCR-signalosome components BTK, MYD88 and NFATC were assessed via real-time quantitative PCR (qPCR). POLR2A was used as a reference gene and fold change was calculated relative to mRNA expression in DMSO-treated cells. mRNA expression of MYD88, BTK and NFATC2 was reduced by 15.8-, 5.8- and 9.9-fold, respectively, compared with DMSO-treated cells. (**b**) Protein expression of MYD88, BTK and NFATC2 was thereafter examined in BCWM.1/IR cells after VLX1570 treatment for 12 h followed by immunoblotting with anti-MYD88, anti-BTK and anti-NFATC2 antibodies. After drug treatment, protein levels of MYD88, BTK and downstream effector NFATC2 (total protein) showed marked reduction. (**c**) WM cells were treated with VLX1570 at indicated concentrations for 12 h and the status of activated NF-κB was assessed by probing for cytoplasmic and nuclear fractions of phosphorylated-NF-κB (p-NFκB/p-p65). VLX1570 treatment reduced nuclear translocation of p-NFκB in all WM cell lines tested (one representative model shown). (**d**) CXCR4 mRNA levels were assessed in BCWM.1/IR cells in a manner similar to (**a**) and were found to be reduced by 3.9-fold relative to DMSO-treated cells. (**e**) Decrease in CXCR4 was further affirmed by assessing expression of the surface receptor. BCWM.1/IR cells were treated with DMSO or VLX1570 (250 nm) for 6 h and incubated with anti-CD184 antibody followed by flow cytometry analysis. Histogram demonstrates CXCR4 receptor expression was reduced by 21–28% in VLX1570-treated cells relative to DMSO-treated counterparts (one representative histogram shown). Black line represents isotype control (DMSO-treated), green line indicates CXCR4 in DMSO-treated cells and red line indicates its expression in VLX1570-treated cells.

The 26 S proteasome regulates bioavailability of several kinases and those of the TEC family, which includes BTK.^38^ Bortezomib has been shown to downregulate the expression of BTK as well as other BCR-signalosome components such as MYD88.^38, 39^ We addressed the question of whether this phenomenon could be similarly elicited by blocking the DUB (USP14/UCHL5) machinery (USP14/UCHL5) of the 19 S proteasome with VLX1570. As anticipated, exposure of BCWM.1/IR cells to VLX1570 (3 h treatment) resulted in decreased transcription of BTK and MYD88 along with their downstream effector NFATC2 (Figure 5a). This was then validated by examining the corresponding protein levels, where immunoblot analysis conducted on VLX1570-treated BCWM.1/IR cells demonstrated reduced expression of BTK, MYD88 and NFATC2. BCR signaling ultimately terminates in the activation of nuclear factor-κB (NF-κB).^40^ Thus, we interrogated the impact of VLX1570 on NF-κB and by using western blot analysis we observed that VLX1570 decreased the nuclear translocation of phosphorylated-NF-κB (p-p65) (Figure 5c, one representative cell line shown).

WM cells are known to rely on CXCR4 signaling for homing into the bone marrow, and interestingly,^41^ CXCR4 receptor expression is regulated by USP14.^20^ As such, we examined its expression in WM cells after treatment with VLX1570 (250 nm, 3 h treatment) and observed not only decreased CXCR4 mRNA but also reduced surface receptor expression (Figures 5d and e). Taken in concert, these analyses reveal for the first time that proteasome inhibition at the 19 S lid alters survival-promoting BCR signaling and chemokine receptor (CXCR4) availability, particularly in IR WM cells.

### WM-bearing mice treated with VLX1570 show significant reduction in tumor burden and increased survival

**Figure 6:**
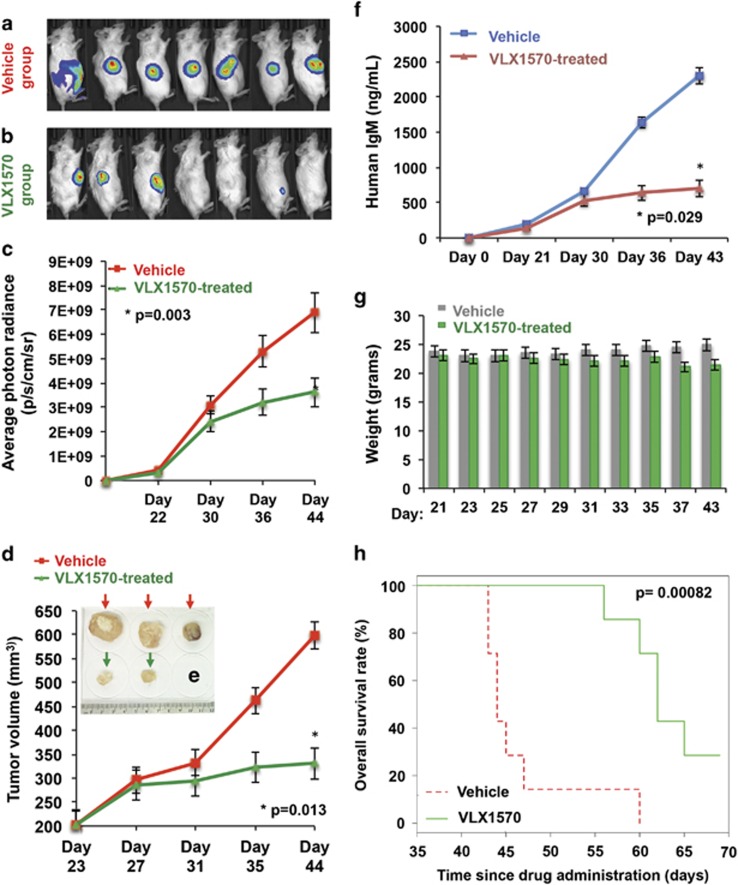
Anti-WM activity of VLX1570 in a xenograft model of aggressive WM. Luc-RPCI-WM1 cells, 1 × 10^6^ were subcutaneously injected into 14 mice and tumors were allowed to grow till bioluminescent signal was observed by IVIS imaging. On day 22, mice were randomized into two groups (*n*=7 each), with one group receiving vehicle (Cremaphor, PEG, Tween) and the other receiving VLX1570 at 4.4 mg/kg via intraperitoneal injection every alternate day for 20 days. (**a**) On day 44 post-tumor implantation, VLX1570-treated mice showed tumor regression or significantly slower rate of tumor growth compared with (**b**) vehicle-treated mice. (**c**) Tumor volume was quantified by bioluminescent photon radiance as well as (**d**) direct caliper measurements and was significantly lower in VLX1570-treated mice by ~day 35 post-tumor implantation (after eight treatments). Data shown are mean±s.d. (*P*=0.003<0.013). (**e**) When tumor volume reached beyond 2000 mm^3^, mice were killed and tumors harvested, which revealed larger tumor size in vehicle-treated mice vs VLX1570-treated mice. (**f**) A third method of quantifying tumor burden was used by measuring human IgM secreted in the mouse serum from the xenografted Luc-RPCI-WM1 cells, which was significantly lower in VLX1570-treated mice (*P*=0.029). (**g**) Body weight measurements were conducted everyday and showed insignificant weight loss in some mice by ~day 37, with the overall (VLX1570) treatment regimen tolerated by mice. (**h**) Kaplan–Meir analysis was conducted to determine overall survival and showed that mice treated with VLX1570 survived an average of ~25 days longer than mice treated with vehicle alone (*P*=0.00082).

Although b-AP15 has been examined in murine models of various cancers^30^ including MM,^33^ the *in vivo* activity of its lead optimized analog, VLX1570, has been described in only two disease models thus far.^42, 43^ Thus, using our murine xenograft model of WM,^44^ we administered VLX1570 via intraperitoneal injection to WM-bearing mice. Xenografted tumors were allowed to grow in size until detection by bioluminescent imaging confirmed congruent tumors (~200 mm^3^) in all mice. Thereafter, mice were randomized into two groups, receiving either vehicle or VLX1570 (4.4 mg/kg) every alternate day. After 21 days of treatment, compared with the vehicle-treated group, we observed a significant reduction in tumor volume and growth as detected by both bioluminescent radiance (Figures 6a–c) and direct caliper measurements (Figures 6d and e). As a secondary systemic marker of response, we measured human immunoglobulin M (IgM), which is secreted by the xenotransplanted WM cells into the mice sera. Significantly lower human IgM levels were noted in VLX1570-treated mice versus those in the vehicle-treated group (Figure 6f). VLX1570 treatment was well tolerated with insignificant weight loss observed in drug-treated mice by day 37 and no other signs of systemic toxicity (Figure 6g). Importantly, a significant survival advantage in mice treated with VLX1570 was noted as compared with mice receiving vehicle alone (median survival, 62 vs 44 days, respectively, *P*=0.0008) (Figure 6h).

**Figure 7:**
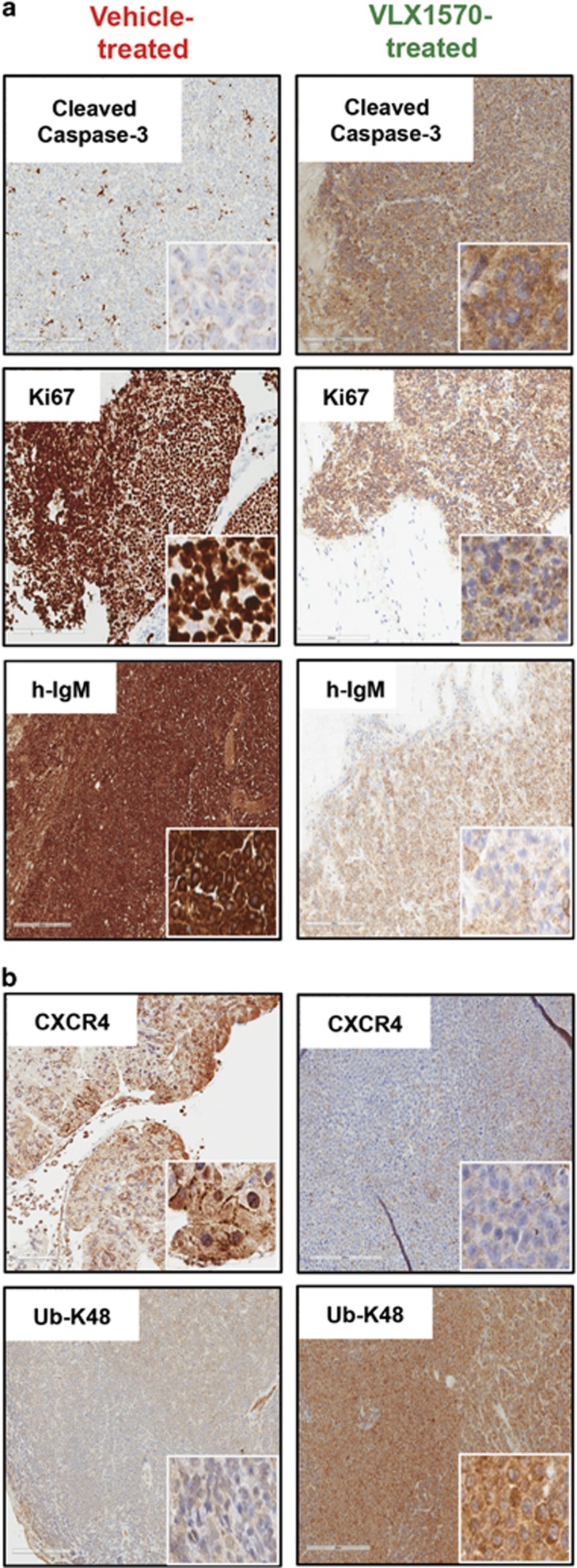
Pharmacodynamic effects of VLX1570 in drug-treated mice tumor tissue. Immunohistochemistry (IHC) analysis was conducted on tumor tissue sections harvested from mice treated with either vehicle or VLX1570. Staining with appropriate antibodies showed (**a**) notably increased staining intensity of cleaved caspase-3 (apoptosis marker), reduced staining intensity for Ki-67 (proliferation marker) and h-IgM (human IgM, tumor burden marker) in VLX1570-treated mice tissues compared with vehicle-treated. (**b**) USP14 substrate, CXCR4 (migration/metastasis marker) as well as Ub-K-48-linked protein conjugates was observed in mice treated with VLX1570 relative to the vehicle group.

Pharmacodynamic effects of therapy were studied using immunohistochemistry analysis on mice tumor tissues and showed increased apoptosis (cleaved caspase-3) as well as decrease in WM cell proliferation (Ki-67) and secretion of human IgM in VLX1570-treated mice (Figure 7a). *In vivo* target engagement was assessed in a more specific manner by staining for K-48-linked ubiquitinated protein accumulation and CXCR4, which were increased and decreased, respectively, in mice treated with VLX1570 relative to vehicle-treated mice (Figure 7b). These data provide first evidence on the *in vivo* antitumor activity profile, safety and survival benefit associated with VLX1570 treatment of WM-xenografted mice, as well as the drugs ability to modulate proteins critical for WM cell survival.

### VLX1570 and ibrutinib display synergistic activity in decreasing IR WM cell viability

**Figure 8:**
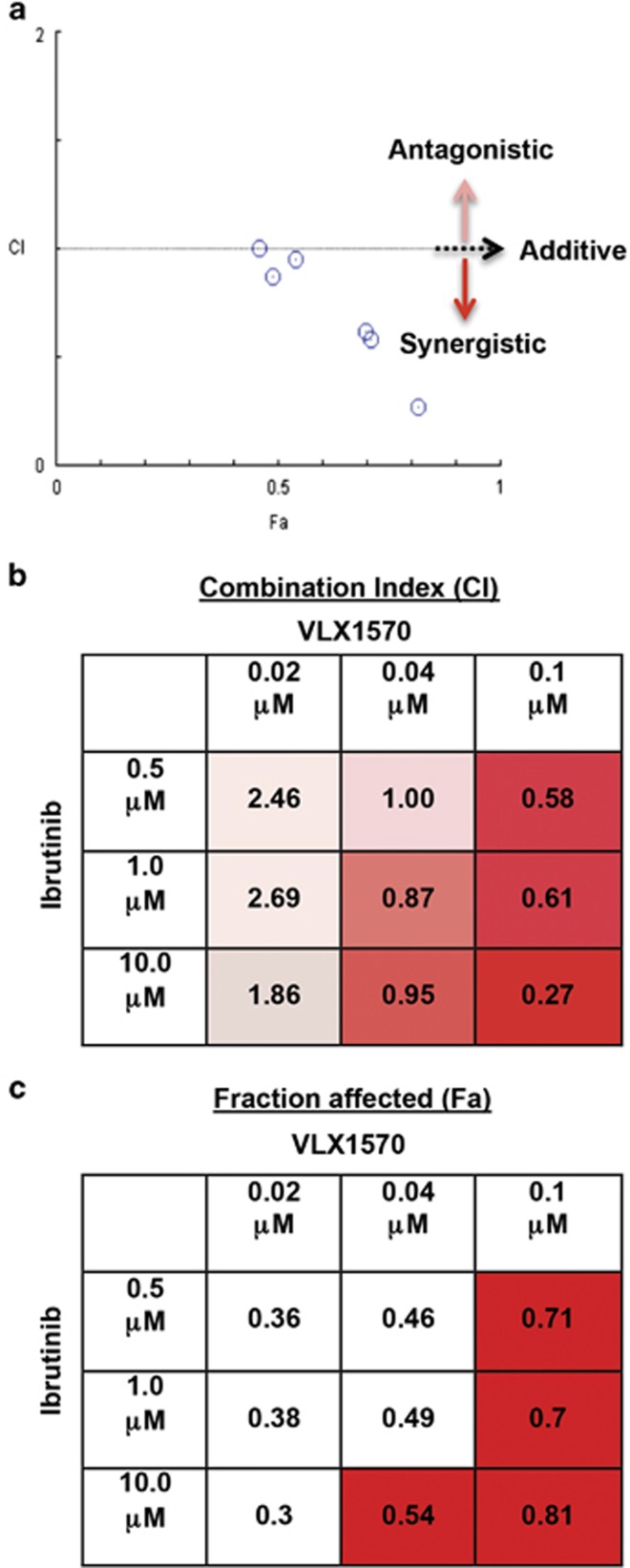
Combination of VLX1570 and ibrutinib is synergistic in IR WM cells. RPCI-WM1/IR cells were treated with VLX1570, ibrutinib or VLX1570+ibrutinib or DMSO for 24 h and assessed for viability by the CellTiter Glo assay (Promega Corp.). (**a**) Isobologram analysis demonstrated synergistic cytotoxic activity of VLX1570+ibrutinib in 5/9 combinations (CI: <1). CI: >1=antagonism and CI=1 signifies additivity. (**b**) The CI values used to create the isobologram are presented, where boxes shaded from pink to red signify antagonistic–synergistic reactions. (**c**) Similarly, the fraction of cells affected (Fa) that were used to create the isobologram are presented with red boxes highlighted to demonstrate the drug concentrations where ⩾50% of cells (Fa 0.54–0.81) were affected by the combination.

Compared with monotherapy, drug combination therapy is known to yield improved antitumor activity as well as counter drug resistance. We addressed the question of whether combining VLX1570 with another agent could induce cytotoxicity in IR WM cells and overcome their insensitivity to single-agent ibrutinib. We treated RPCI-WM1/IR cells with different concentrations of VLX1570+ibrutinib for 24 h followed by measurement of cell viability. This combination induced a synergistic reaction (Figure 8a) in five out of nine dose permutations tested (median CI: 0.3; range: 0.2–0.9) (Figure 8b) and significantly reduced tumor cell viability with the fraction of affected cells (Fa) ranging between 0.3 and 0.81 (30–81% cell death; median Fa: 0.49) (Figure 8c). These data suggest the ability of VLX1570 and ibrutinib to successfully synergize at various combinatorial concentrations to induce optimal cell death in WM cells that are resistant to ibrutinib monotherapy.

## Discussion

There are ~98 DUBs encoded by the human genome, which are distributed into six families based on sequence and structural similarities.^45^ The 19 S regulatory particle of the proteasome harbors three DUBs, each exhibiting notable differences in homology.^17^ Of the three, USP14 and UCHL5 reversibly associate with the proteasome, whereas the third, RPN11/POH1, is an intrinsic component of the lid subcomplex of the 19 S cap.^18^ The role of USP14 and UCHL5 in maintaining cancer cell viability and providing growth advantage is increasingly being recognized in a variety of cancers, including hematologic malignancies.^30^ Although the precise molecular contributions made by these two DUBs towards tumor cell survival continue to be studied,^34, 46, 47, 48, 49, 50^ efforts to target their dysregulated (and often increased) activity are underway, notably in MM and WM.^23, 30^ Herein, we extend our initial observations that USP14 and UCHL5 are highly expressed in drug-resistant WM tumor cells including those from WM patients as compared with PBMCs or resident bone marrow cells from healthy donors. Affirming their functional significance, we observed that targeted codisruption of both DUBs with the first in class USP14/UCHL5 inhibitor VLX1570 leads to robust *in vitro*, *ex vivo* and *in vivo* WM-specific apoptotic cell death.

VLX1570 was developed from b-AP15, which was identified in a screen for compounds that induce the lysosomal apoptosis pathway.^30^ To make b-AP15 (a piperdine-based chemical) suitable for administration in humans, med-chem techniques were used to create several novel derivatives. It was found that analogs containing a central azapene ring possessed greater biological activity, of which VLX1570 emerged as the most potent.^31^ Our *in silico* docking analysis uncovered potential structural differences between the two molecules (b-AP15 and VLX1570) that account for the improved activity of VLX1570. VLX1570 maintains better pi-pi-cloud planarity with nearest neighbors that exhibit pi-cloud orbital mechanics and its overall position has a better pocket occupancy than b-AP15. From energy assessment of its structure (minimization and foldX), the dihedral and thiol (–SH) arrangement appear more stable in VLX1570. Lesser water would be allowed to penetrate into the region where the alkane/alkene from the drug abuts to the protein's (USP14 and UCHL5) hydrophobic region of the binding pocket. Indeed, we observed enhanced performance of VLX1570 in virtually all cell-based assays (apoptosis, MOMP and protein modulation) where lower concentrations (~250 nm) sufficiently induced changes that were nearly equivalent to when twice the concentration of b-AP15 (~500–1000 nm) was used in identical assays for the same duration. Notably, this phenomenon was preserved in WM cells resistant to bortezomib or ibrutinib.

The activity profile of VLX1570 in BR WM cells is intriguing and the exact nature of why these cells retain sensitivity to proteasome blockade at the 19 S cap but resist downstream 20 S core inhibition remains enigmatic. One potential reason for this differential activity may be because of the type of polyubiquitinated proteins that accumulate in the cytosol of VLX1570- vs bortezomib-treated cells. Our own comparative analysis herein, using the two drugs side by side and measuring ubiquitin-conjugated protein in BR WM cells, appeared consistent with prior reports that USP14/UCHL5 inhibition (with b-AP15) produces buildup of higher-molecular-weight (⩾400 kDa) proteins than does bortezomib (~260–290 kDa).^30, 33^ It is conceivable that buildup of these higher kDa proteins after VLX1570 treatment overload the cells clearance capacity in a much shorter duration (within 3 h), thereby committing the cells towards apoptosis—an effect that is not achieved by the same concentration/time exposure with bortezomib.

The activity of VLX1570 was also observable in IR WM cells, an encouraging and highly desirable property for a new drug (being developed for WM) to possess. Notably, these IR WM cells lack both BTK^C481S^ or CXCR4^WHIM^ mutations and display ~5-fold apoptosis resistance towards ibrutinib.^26, 27, 51^ Although 20 S PI such as bortezomib have been shown to modulate levels of TEC family kinases, including BTK, ITK, Bmx and Tec, as well as other BCR-signalosome components such as MYD88,^38, 39^ we questioned whether this effect would be similar with 19 S (USP14/UCHL5) inhibition. As anticipated, coinhibition of the 19 S DUBs reduced the transcriptional and translational expression of BTK and MYD88. Both are actively involved in transmission of signaling towards their end effectors NFATC2 and NF-κB,^40^ levels of which are decreased following VLX1570 treatment. Although we examined NF-κB translocation as a downstream function of BCR signaling, it may be possible that the inverse holds true where BTK levels are reduced owing to downregulation of NF-κB by VLX1570. NF-κB signaling regulates BTK expression and Yu *et al.*^38^ have shown that BTK uses a positive autoregulatory feedback mechanism to stimulate transcription from its own promoter via NF-κB. The effect of NF-κB downregulation on MYD88 is more elusive. However, a screen for genes^52^ that when perturbed result in decreased MYD88 transcription revealed both *Rela* and *Relb* (NF-κB components) as two of several genes that negatively regulate MYD88 levels (Supplementary Table 5). Ongoing analysis will delineate the significance of this finding and to the best of our knowledge we are first to show this phenomenon occuring in malignant B cells. Overall, these mechanisms are in alignment with our previous findings on USP14/UCHL5 inhibition and its direct downregulation of NF-κB gene activation.^23^ The reduced expression of CXCR4 is also of interest and its deubiquitination by USP14 is necessary for the chemokine receptors degradation and bioavailability.^20^ This would explain decreased surface expression; however, we also observed a significant decrease in USP14 mRNA levels. A plausible explanation for this event once again may be linked to NF-κB, where the p50 and p65 subcomplexes bind the CXCR4 gene promoter region and regulate its activation resulting in tumor cell migration.^53^ Yet, whether NF-κB is directly or indirectly responsible for decreasing CXCR4 in malignant B cells remains to be further studied.

To assess the *in vivo* anti-WM capabilities of VLX1570, we used our xenograft murine model of aggressive human WM, using the RPCI-WM1 cell line. RPCI-WM1 was developed from a bortezomib/rituximab refractory WM patient and displays inherent apoptosis resistance towards ibrutinib *in vitro*.^44, 54^ As described in a prior report, robust activity of USP14/UCHL5 inhibition with b-AP15 was observed in delaying the outgrowth of tumor cells in MM-xenotransplanted mice.^33^ Whereas Tian *et al.*^33^ administered b-AP15 at 4 mg/kg on a consecutive day schedule for 14 days, owing to the improved chemistry of VLX1570, we opted for a similar dose but on an alternate day schedule consisting of 12 treatments. This regimen was well tolerated by the mice and systemic drug activity was observed by several parameters including reduction of circulating human IgM in mice sera, decreased expression of CXCR4 and K-48-linked ubiquitinated protein levels, with the latter two attesting to *in vivo* target specificity of VLX1570. In concert, these data provide further validation of USP14 and UCHL5 as bonafide targets in WM and the ability of VLX1570 to reduce WM cell growth in tumor bearing mice while extending their survival.

Our preclinical analyses demonstrate the robust activity of VLX1570 alone as well as in combination with another therapeutic. We opted to test pharmacologic synergy of VLX1570 with the standard of WM care agent ibrutinib as it stood to reason not only from a clinical developmental standpoint but also from a biological perspective. Each agent effectively disrupts separate modules of a common axis that results in downregulation of NF-κB; thus, we hypothesized that simultaneous disruption of this oncogenic axis at the 19 S proteasome (via VLX1570) and directly at the BCR/MY88_L265P_ module (via ibrutinib) would result in enhanced tumor kill. A second interest was to determine if this combination would be effective in WM cells resistant to ibrutinib. Our experiments demonstrate synergy between VLX1570 and ibrutinib, supporting the potential benefit of adding VLX1570 to an ibrutinib-based regimen to potentially delay (or prevent) onset of resistance to ibrutinib as well as benefit existing patients already on ibrutinib but which begin to exhibit insensitivity to the BTK inhibitor.

In summary, we present compelling data on the targetability of the 19 S proteasome-associated DUBs, USP14 and UCHL5 with the use of VLX1570, which induces tumor-specific cell death, particularly in WM cells resistant to bortezomib or ibrutinib. The proteasome remains a well-validated, yet underdeveloped target in oncology. VLX1570 is a first in class USP14/UCHL5-specific inhibitor, which modulates biocellular pathways (i.e. BCR signaling components) that are essential for WM cell survival and whose cellular effects are in many ways distinct from 20 S PI such as bortezomib. *In vitro*, *in vivo* and in silico data presented in this report lay the framework for further development of VLX1570 for clinical use in patients with WM.

## References

[bib1] Treon SP, Tripsas CK, Meid K, Warren D, Varma G, Green R et al. Ibrutinib in previously treated Waldenstrom's macroglobulinemia. N Engl J Med 2015; 372: 1430–1440. 25853747 10.1056/NEJMoa1501548

[bib2] Cao Y, Hunter ZR, Liu X, Xu L, Yang G, Chen J et al. The WHIM-like CXCR4(S338X) somatic mutation activates AKT and ERK, and promotes resistance to ibrutinib and other agents used in the treatment of Waldenstrom's macroglobulinemia. Leukemia 2015; 29: 169–176. 24912431 10.1038/leu.2014.187

[bib3] Young RM, Staudt LM. Ibrutinib treatment of CLL: the cancer fights back. Cancer Cell 2014; 26: 11–13. 25026208 10.1016/j.ccr.2014.06.023 PMC4199743

[bib4] Woyach JA, Furman RR, Liu TM, Ozer HG, Zapatka M, Ruppert AS et al. Resistance mechanisms for the Bruton's tyrosine kinase inhibitor ibrutinib. N Engl J Med 2014; 370: 2286–2294. 24869598 10.1056/NEJMoa1400029 PMC4144824

[bib5] Furman RR, Cheng S, Lu P, Setty M, Perez AR, Guo A et al. Ibrutinib resistance in chronic lymphocytic leukemia. N Engl J Med 2014; 370: 2352–2354. 24869597 10.1056/NEJMc1402716 PMC4512173

[bib6] Chiron D, Di Liberto M, Martin P, Huang X, Sharman J, Blecua P et al. Cell-cycle reprogramming for PI3K inhibition overrides a relapse-specific C481S BTK mutation revealed by longitudinal functional genomics in mantle cell lymphoma. Cancer Discov 2014; 4: 1022–1035. 25082755 10.1158/2159-8290.CD-14-0098 PMC4155003

[bib7] Cao Y, Hunter ZR, Liu X, Xu L, Yang G, Chen J et al. CXCR4 WHIM-like frameshift and nonsense mutations promote ibrutinib resistance but do not supplant MYD88-directed survival signalling in Waldenstrom macroglobulinaemia cells. Br J Haematol 2014 2015; 168: 701–707. 10.1111/bjh.13200 25371371

[bib8] Jain P, Keating M, Wierda W, Estrov Z, Ferrajoli A, Jain N et al. Outcomes of patients with chronic lymphocytic leukemia (CLL) after discontinuing ibrutinib. Blood 2015; 125: 2062–2067. 25573991 10.1182/blood-2014-09-603670 PMC4467871

[bib9] Treon SP, Xu L, Hunter Z. MYD88 mutations and response to ibrutinib in Waldenstrom's macroglobulinemia. N Engl J Med 2015; 373: 584–586. 10.1056/NEJMc1506192 26244327

[bib10] Adams J. The proteasome: structure, function, and role in the cell. Cancer Treat Rev 2003; 29(Suppl 1): 3–9. 10.1016/s0305-7372(03)00081-1 12738238

[bib11] Melino G. Discovery of the ubiquitin proteasome system and its involvement in apoptosis. Cell Death Differ 2005; 12: 1155–1157. 16094390 10.1038/sj.cdd.4401740

[bib12] Mato AR, Feldman T, Goy A. Proteasome inhibition and combination therapy for non-Hodgkin's lymphoma: from bench to bedside. Oncologist 2012; 17: 694–707. 22566373 10.1634/theoncologist.2011-0341 PMC3360909

[bib13] Sekhar J, Sanfilippo K, Zhang Q, Trinkaus K, Vij R, Morgensztern D. Waldenstrom macroglobulinemia: a Surveillance, Epidemiology, and End Results database review from 1988 to 2005. Leuk Lymphoma 2012; 53: 1625–1626. 22239669 10.3109/10428194.2012.656103

[bib14] Treon SP, Hunter ZR, Matous J, Joyce RM, Mannion B, Advani R et al. Multicenter clinical trial of bortezomib in relapsed/refractory Waldenstrom's macroglobulinemia: results of WMCTG Trial 03-248. Clin Cancer Res 2007; 13: 3320–3325. 17545538 10.1158/1078-0432.CCR-06-2511

[bib15] Ghobrial IM, Hong F, Padmanabhan S, Badros A, Rourke M, Leduc R et al. Phase II trial of weekly bortezomib in combination with rituximab in relapsed or relapsed and refractory Waldenstrom macroglobulinemia. J Clin Oncol 2010; 28: 1422–1428. 20142586 10.1200/JCO.2009.25.3237 PMC2834499

[bib16] Dimopoulos MA, Terpos E, Kastritis E. Proteasome inhibitor therapy for Waldenstrom's macroglobulinemia. Clin Lymphoma Myeloma Leuk 2013; 13: 235–237. 23562304 10.1016/j.clml.2013.02.014

[bib17] D'Arcy P, Linder S. Proteasome deubiquitinases as novel targets for cancer therapy. Int J Biochem Cell Biol 2012; 44: 1729–1738. 22819849 10.1016/j.biocel.2012.07.011

[bib18] Pathare GR, Nagy I, Sledz P, Anderson DJ, Zhou HJ, Pardon E et al. Crystal structure of the proteasomal deubiquitylation module Rpn8–Rpn11. Proc Natl Acad Sci USA 2014; 111: 2984–2989. 24516147 10.1073/pnas.1400546111 PMC3939901

[bib19] Chen Z, Niu X, Li Z, Yu Y, Ye X, Lu S et al. Effect of ubiquitin carboxy-terminal hydrolase 37 on apoptotic in A549 cells. Cell Biochem Funct 2011; 29: 142–148. 21287580 10.1002/cbf.1734

[bib20] Mines MA, Goodwin JS, Limbird LE, Cui FF, Fan GH. Deubiquitination of CXCR4 by USP14 is critical for both CXCL12-induced CXCR4 degradation and chemotaxis but not ERK ativation. J Biol Chem 2009; 284: 5742–5752. 19106094 10.1074/jbc.M808507200 PMC2645827

[bib21] Rolen U, Kobzeva V, Gasparjan N, Ovaa H, Winberg G, Kisseljov F et al. Activity profiling of deubiquitinating enzymes in cervical carcinoma biopsies and cell lines. Mol Carcinogen 2006; 45: 260–269. 10.1002/mc.20177 16402389

[bib22] Shinji S, Naito Z, Ishiwata S, Ishiwata T, Tanaka N, Furukawa K et al. Ubiquitin-specific protease 14 expression in colorectal cancer is associated with liver and lymph node metastases. Oncol Rep 2006; 15: 539–543. 16465409

[bib23] Chitta K, Paulus A, Akhtar S, Blake MK, Caulfield TR, Novak AJ et al. Targeted inhibition of the deubiquitinating enzymes, USP14 and UCHL5, induces proteotoxic stress and apoptosis in Waldenstrom macroglobulinaemia tumour cells. Br J Haematol 2015; 169: 377–390. 25691154 10.1111/bjh.13304 PMC4846423

[bib24] Chitta K, Miles KM, Ghoshal P, Stein L, Coleman M, Furman RR et al. AT-101 induces apoptosis Waldenstrom macroglobulinemia cells resistant to bortezomib. Blood 2009; 114: 2861.19636061

[bib25] Paulus A, Chitta K, Akhtar S, Personett D, Miller KC, Thompson KJ et al. AT-101 downregulates BCL2 and MCL1 and potentiates the cytotoxic effects of lenalidomide and dexamethasone in preclinical models of multiple myeloma and Waldenstrom macroglobulinaemia. Br J Haematol 2014; 164: 352–365. 24236538 10.1111/bjh.12633 PMC4406280

[bib26] Chitta KS, Paulus A, Kuranz-Blake M, Akhtar S, Novak AJ, Ansell SM et al. Acquired *in vitro* resistance to ibrutinib is associated with transcriptional re-programming and sustained survival signaling in Waldenströms macroglobulinemia and mantle cell lymphoma, independent of BTK Cys481 mutation. Blood 2014; 124: 2250.

[bib27] Chitta K, Paulus A, Caulfield TR, Akhtar S, Blake MK, Ailawadhi S et al. Nimbolide targets BCL2 and induces apoptosis in preclinical models of Waldenstroms macroglobulinemia. Blood Cancer J 2014; 4: e260. 25382610 10.1038/bcj.2014.74 PMC5424099

[bib28] Chitta KS, Khan AN, Ersing N, Swaika A, Masood A, Paulus A et al. Neem leaf extract induces cell death by apoptosis and autophagy in B-chronic lymphocytic leukemia cells. Leuk Lymphoma 2014; 55: 652–661. 23721511 10.3109/10428194.2013.807927

[bib29] Chou TC, Talalay P. Quantitative analysis of dose–effect relationships: the combined effects of multiple drugs or enzyme inhibitors. Adv Enzyme Regul 1984; 22: 27–55. 6382953 10.1016/0065-2571(84)90007-4

[bib30] D'Arcy P, Brnjic S, Olofsson MH, Fryknas M, Lindsten K, De Cesare M et al. Inhibition of proteasome deubiquitinating activity as a new cancer therapy. Nat Med 2011; 17: 1636–1640. 22057347 10.1038/nm.2536

[bib31] Wang X, D'Arcy P, Caulfield TR, Paulus A, Chitta K, Mohanty C et al. Synthesis and evaluation of derivatives of the proteasome deubiquitinase inhibitor b-AP15. Chem Biol Drug Des 2015; 86: 1036–1048. 25854145 10.1111/cbdd.12571 PMC4846425

[bib32] Drexler HG, Chen S, Macleod RA. Would the real Waldenstrom cell line please stand up? Leuk Lymphoma 2013; 54: 224–226. 22950966 10.3109/10428194.2012.727418

[bib33] Tian Z, D'Arcy P, Wang X, Ray A, Tai YT, Hu Y et al. A novel small molecule inhibitor of deubiquitylating enzyme USP14 and UCHL5 induces apoptosis in multiple myeloma and overcomes bortezomib resistance. Blood 2014; 123: 706–716. 24319254 10.1182/blood-2013-05-500033 PMC3907756

[bib34] Brnjic S, Mazurkiewicz M, Fryknas M, Sun C, Zhang X, Larsson R et al. Induction of tumor cell apoptosis by a proteasome deubiquitinase inhibitor is associated with oxidative stress. Antioxidants Redox Signal 2014; 21: 2271–2285. 10.1089/ars.2013.5322 PMC4241954 24011031

[bib35] Ron D, Walter P. Signal integration in the endoplasmic reticulum unfolded protein response. Nat Rev Mol Cell Biol 2007; 8: 519–529. 17565364 10.1038/nrm2199

[bib36] Kroemer G, Galluzzi L, Brenner C. Mitochondrial membrane permeabilization in cell death. Physiol Rev 2007; 87: 99–163. 17237344 10.1152/physrev.00013.2006

[bib37] Gupta S, Cuffe L, Szegezdi E, Logue SE, Neary C, Healy S et al. Mechanisms of ER stress-mediated mitochondrial membrane permeabilization. Int J Cell Biol 2010; 2010: 170–215. 10.1155/2010/170215 PMC2821636 20169117

[bib38] Yu L, Mohamed AJ, Simonson OE, Vargas L, Blomberg KE, Bjorkstrand B et al. Proteasome-dependent autoregulation of Bruton tyrosine kinase (Btk) promoter via NF-kappaB. Blood 2008; 111: 4617–4626. 18292289 10.1182/blood-2007-10-121137

[bib39] Nencioni A, Schwarzenberg K, Brauer KM, Schmidt SM, Ballestrero A, Grunebach F et al. Proteasome inhibitor bortezomib modulates TLR4-induced dendritic cell activation. Blood 2006; 108: 551–558. 16537813 10.1182/blood-2005-08-3494

[bib40] Young RM, Staudt LM. Targeting pathological B cell receptor signalling in lymphoid malignancies. Nat Rev Drug Discov 2013; 12: 229–243. 23449308 10.1038/nrd3937 PMC7595252

[bib41] Ngo HT, Leleu X, Lee J, Jia X, Melhem M, Runnels J et al. SDF-1/CXCR4 and VLA-4 interaction regulates homing in Waldenstrom macroglobulinemia. Blood 2008; 112: 150–158. 18448868 10.1182/blood-2007-12-129395 PMC2435685

[bib42] Shukla N, Somwar R, Smith RS, Ambati S, Munoz S, Merchant M et al. Proteasome addiction defined in ewing sarcoma is effectively targeted by a novel class of 19 S proteasome inhibitors. Cancer Res 2016; 76: 4525–4534. 27256563 10.1158/0008-5472.CAN-16-1040 PMC5484002

[bib43] Wang X, Mazurkiewicz M, Hillert EK, Olofsson MH, Pierrou S, Hillertz P et al. The proteasome deubiquitinase inhibitor VLX1570 shows selectivity for ubiquitin-specific protease-14 and induces apoptosis of multiple myeloma cells. Scientific Rep 2016; 6: 26979. 10.1038/srep26979 PMC4893612 27264969

[bib44] Chitta KS, Paulus A, Ailawadhi S, Foster BA, Moser MT, Starostik P et al. Development and characterization of a novel human Waldenstrom macroglobulinemia cell line: RPCI-WM1, Roswell Park Cancer Institute—Waldenstrom Mamroglobulinemia 1. Leuk Lymphoma 2013; 54: 387–396. 22812491 10.3109/10428194.2012.713481 PMC4406272

[bib45] Fraile JM, Quesada V, Rodriguez D, Freije JM, Lopez-Otin C. Deubiquitinases in cancer: new functions and therapeutic options. Oncogene 2012; 31: 2373–2388. 21996736 10.1038/onc.2011.443

[bib46] Vogel RI, Coughlin K, Scotti A, Iizuka Y, Anchoori R, Roden RB et al. Simultaneous inhibition of deubiquitinating enzymes (DUBs) and autophagy synergistically kills breast cancer cells. Oncotarget 2015; 6: 4159–4170. 25784654 10.18632/oncotarget.2904 PMC4414179

[bib47] Sarhan D, D'Arcy P, Lundqvist A. Regulation of TRAIL-receptor expression by the ubiquitin-proteasome system. Int J Mol Sci 2014; 15: 18557–18573. 25318057 10.3390/ijms151018557 PMC4227232

[bib48] Wang X, Stafford W, Mazurkiewicz M, Fryknas M, Brjnic S, Zhang X et al. The 19 S deubiquitinase inhibitor b-AP15 is enriched in cells and elicits rapid commitment to cell death. Mol Pharmacol 2014; 85: 932–945. 24714215 10.1124/mol.113.091322

[bib49] Feng X, Holmlund T, Zheng C, Fadeel B. Proapoptotic effects of the novel proteasome inhibitor b-AP15 on multiple myeloma cells and natural killer cells. Exp Hematol 2014; 42: 172–182. 24291587 10.1016/j.exphem.2013.11.010

[bib50] Sarhan D, Wennerberg E, D'Arcy P, Gurajada D, Linder S, Lundqvist A. A novel inhibitor of proteasome deubiquitinating activity renders tumor cells sensitive to TRAIL-mediated apoptosis by natural killer cells and T cells. Cancer Immunol Immunother 2013; 62: 1359–1368. 23689729 10.1007/s00262-013-1439-1 PMC11029014

[bib51] Azab AK, Paulus A, Azab F, Akhtar S, Vali S, Kumar A et al. A novel and personalized method using simulation for predicting effective therapeutics for waldenströms macroglobulinemia. Blood 2014; 124: 3024.

[bib52] Kupershmidt I, Su QJ, Grewal A, Sundaresh S, Halperin I, Flynn J et al. Ontology-based meta-analysis of global collections of high-throughput public data. PLoS One 2010; 5, pii: e13066. 10.1371/journal.pone.0013066 PMC2947508 20927376

[bib53] Helbig G, Christopherson KW II, Bhat-Nakshatri P, Kumar S, Kishimoto H, Miller KD et al. NF-kappaB promotes breast cancer cell migration and metastasis by inducing the expression of the chemokine receptor CXCR4. J Biol Chem 2003; 278: 21631–21638. 12690099 10.1074/jbc.M300609200

[bib54] Paulus A, Akhtar S, Yoon H, Wang X, Blake-Kuranz M, Wallace PK et al. Therapeutic sensitivity of CD20− Waldenströms macroglobulinemia cells is determined by underlying genomic and epigenetic events. Blood 2014; 124: 3115.

