# Supplementary figures and images for: Coinhibition of the deubiquitinating enzymes, USP14 and UCHL5, with VLX1570 is lethal to ibrutinib- or bortezomib-resistant Waldenstrom macroglobulinemia tumor cells
Source: Blood Cancer J. 2016 Nov 4;6(11):e492–. doi: 10.1038/bcj.2016.93 (PMC5148058; doi:10.1038/bcj.2016.93)

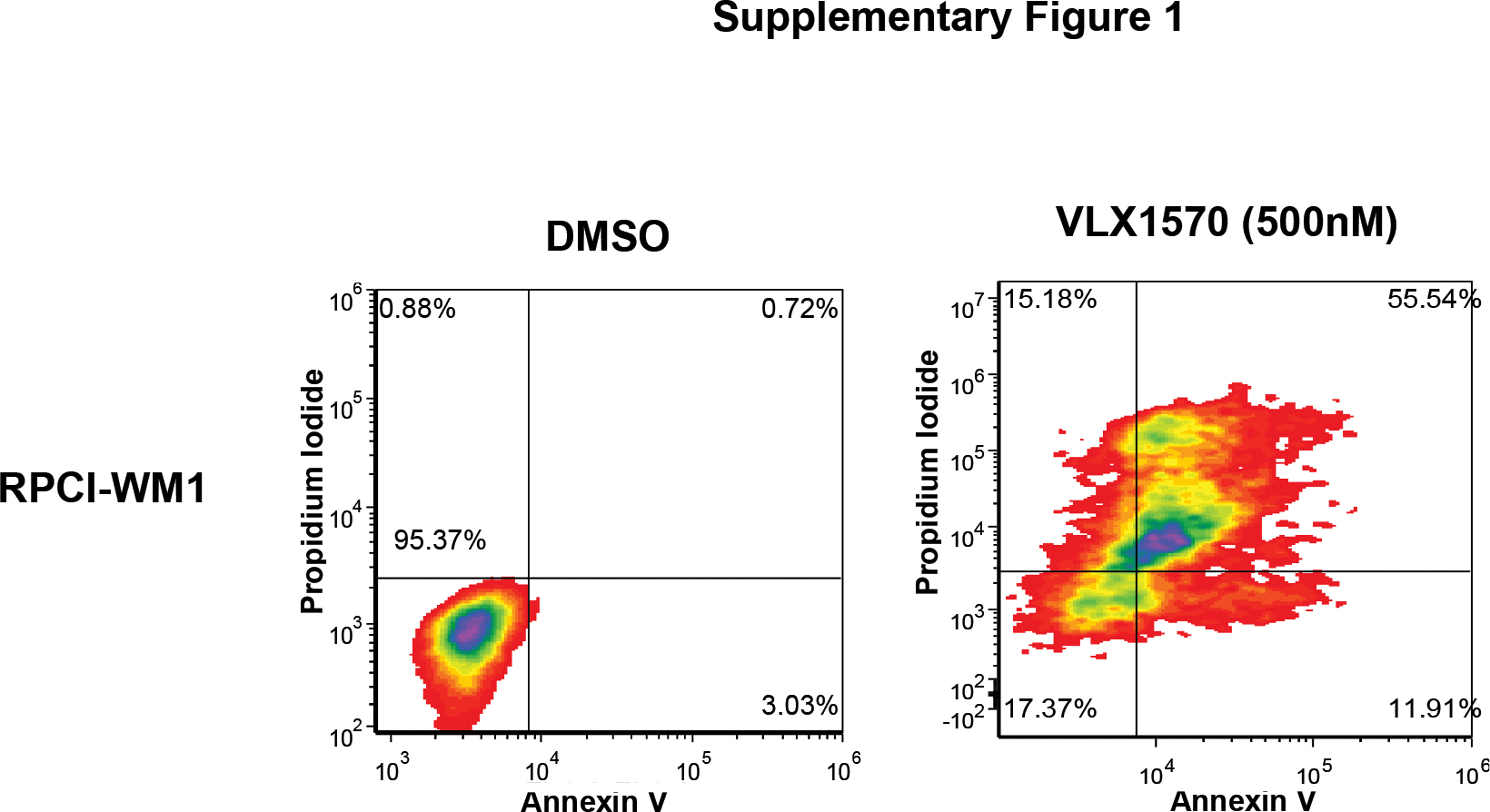

Supplement: Supplementary Figure 1 [file bcj201693x1.tif]

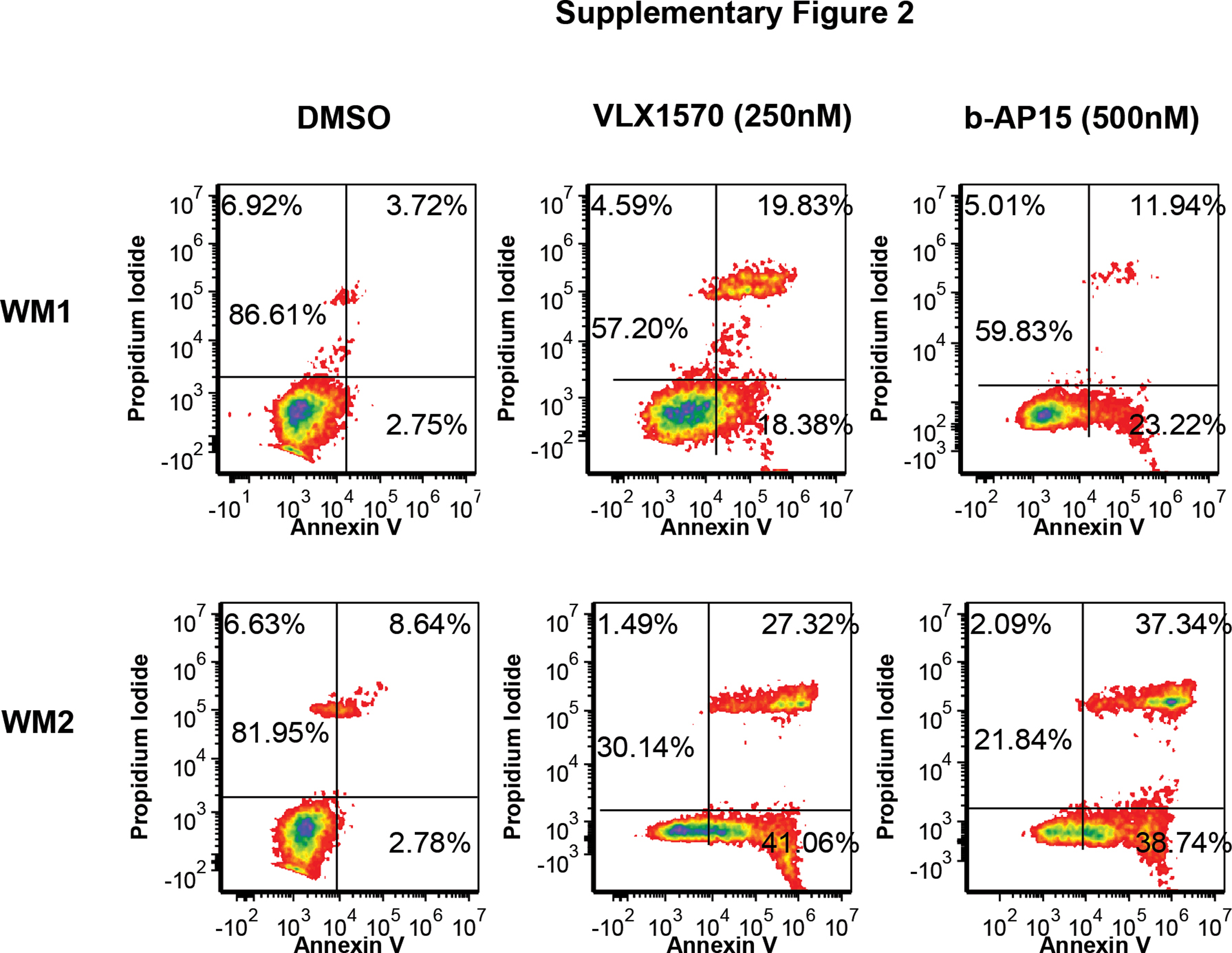

Supplement: Supplementary Figure 2 [file bcj201693x2.tif]

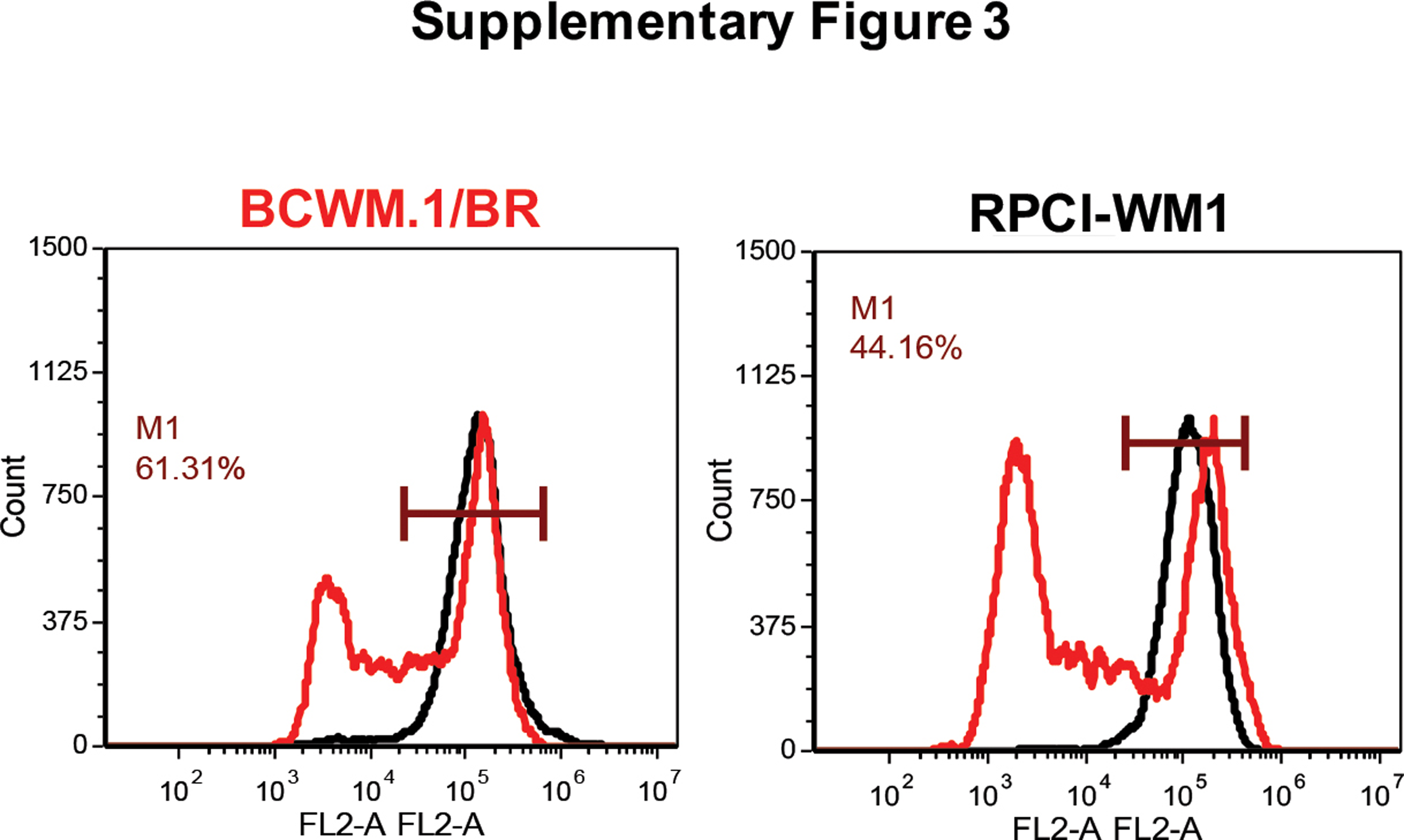

Supplement: Supplementary Figure 3 [file bcj201693x3.tif]
