# Supplementary material for: Coinhibition of the deubiquitinating enzymes, USP14 and UCHL5, with VLX1570 is lethal to ibrutinib- or bortezomib-resistant Waldenstrom macroglobulinemia tumor cells
Source: Blood Cancer J. 2016 Nov 4;6(11):e492–. doi: 10.1038/bcj.2016.93 (PMC5148058; doi:10.1038/bcj.2016.93)
Supplement: Supplementary Tables and Supplemental Figure Legends [file bcj201693x4.docx]

**Supplementary Table 1**

**Sensitivity (EC_50_, nM) of WM cells treated with VLX1570**

| **Cell Lines** | **EC_50_** |
| --- | --- |
| BCWM.1 | 20.2 |
| BCWM-1/BR | 29.96 |
| MWCL-1 | 35.26 |
| RPCI-WM1 | 26.17 |
| RPCI-WM1/BR | 93.59 |

**Supplementary Table 2**

**% Annexin-V staining in WM cell lines after treatment with VLX1570**

|  | **RPCI-WM1** | **BCWM.1** | **BCWM.1/BR** | **RPCI-WM1/BR** | **BCWM.1/IR** |
| --- | --- | --- | --- | --- | --- |
| **DMSO** | 22.90 | 16.80 | 14.17 | 22.60 | 14.7 |
| **VLX1570**  **(100 nM)** | 51.40 | 24.97 | 18.40 | 23.15 | 16.7 |
| **VLX1570**  **(250 nM)** | 72.80 | 53.43 | 30.30 | 39.66 | 23.8 |
| **VLX1570**  **(500 nM)** | 85.70 | 52.00 | 33.34 | 45.44 | 56.0 |
| **p value**  **(DMSO vs.**  **500 nM)** | 0.00003 | 0.00029 | 0.00159 | 0.00160 | 0.01741 |

WM cell lines in red font (BCWM.1/BR and RPCI-WM1/BR) are bortezomib resistant derivatives. WM cell line in blue colored font (BCWM.1/IR) is an ibrutinib resistant variant.

**Supplementary Table 3**

**% Annexin-V staining in primary WM cells from patients and PBMCs from healthy donors treated with VLX1570**

|  | **DMSO** | **VLX1570 250 nM**  **(p-value, relative to DMSO)** | **b-AP15 500 nM**  **(p-value, relative to DMSO)** |
| --- | --- | --- | --- |
| **WM1** | 14.0 | 43.0 (0.00022) | 41.0 (0.00018) |
| **WM2** | 19.0 | 70.0 (0.00009) | 79.0 (0.000036) |
| **PBMC 1** | 7.4 | 9.7 (0.086) | 11.5 (0.040) |
| **PBMC 2** | 13.5 | 12.2 (0.82) | 15.4 (0.072) |

**Supplementary Table 4**

**% MOMP in WM cell lines after treatment with VLX1570**

|  | **RPCI-WM1** | **BCWM.1** | **MWCL-1** | **BCWM.1/BR** |
| --- | --- | --- | --- | --- |
| **DMSO** | 10.58 | 5.43 | 4.15 | 4.33 |
| **VLX1570**  **(100 nM)** | 35.02 | 12.60 | 9.36 | 9.05 |
| **VLX1570**  **(250 nM)** | 71.88 | 48.71 | 9.36 | 32.47 |
| **VLX1570**  **(500 nM)** | 84.23 | 64.84 | 69.56 | 56.42 |
| **p-value**  **(DMSO vs.**  **500 nM)** | 0.000045 | 0.000001 | 0.000046 | 0.00025 |

WM cell line in red font (BCWM.1/BR) is a bortezomib resistant subclone.

**Supplementary Table 5**

**List of genes that regulate MYD88 transcription**

| **Perturbed Gene**  **(Gene knockdown)** | **Data Type** | **# of supporting studies** | **Effect on Query (MYD88)** |
| --- | --- | --- | --- |
| \| **SP3** \| \| --- \| \| | RNA expression | 1 | down-regulated |
|  |  |  |  |
| \| **MYD88** \| \| --- \| \| | RNA expression | 8 | down-regulated |
|  |  |  |  |
| \| **SPO11** \| \| --- \| \| | RNA expression | 2 | down-regulated |
|  |  |  |  |
| \| **DUSP5** \| \| --- \| \| | RNA expression | 1 | down-regulated |
|  |  |  |  |
| \| ***Relb** \| \| --- \| \| | RNA expression | 1 | down-regulated |
|  |  |  |  |
| \| **GJB1** \| \| --- \| \| | RNA expression | 1 | down-regulated |
|  |  |  |  |
| \| **HORMAD1** \| \| --- \| \| | RNA expression | 1 | down-regulated |
|  |  |  |  |
| \| **TMEM173** \| \| --- \| \| | RNA expression | 1 | down-regulated |
|  |  |  |  |
| \| **Wt1** \| \| --- \| \| | RNA expression | 1 | down-regulated |
|  |  |  |  |
| \| **PTPN11** \| \| --- \| \| | RNA expression | 1 | down-regulated |
|  |  |  |  |
| \| ***Rela** \| \| --- \| \| | RNA expression | 1 | down-regulated |
|  |  |  |  |
| \| **STK4** \| \| --- \| \| | RNA expression | 1 | down-regulated |
|  |  |  |  |
| \| **TBX21** \| \| --- \| \| | RNA expression | 1 | down-regulated |
|  |  |  |  |
| \| **RARG** \| \| --- \| \| | RNA expression | 2 | down-regulated |
|  |  |  |  |
| \| **VAV3** \| \| --- \| \| | RNA expression | 1 | down-regulated |
|  |  |  |  |
| \| **IRF8** \| \| --- \| \| | RNA expression | 1 | down-regulated |
|  |  |  |  |
| \| **CHD1** \| \| --- \| \| | RNA expression | \| 1 \| \| --- \| \| | down-regulated |
|  |  |  |  |
| \| **VAV2** \| \| --- \| \| | RNA expression | 1 | down-regulated |
|  |  |  |  |

* Rela and Relb are NFkB components.

Table generated from data curated by Illumina NextBio Research platform (Gene Knockdown Atlas).^1^

Original data source for *Rela and Relb perturbation experiments: Agnes et al, Differential RelA- and RelB-dependent gene transcription in LTbetaR-stimulated mouse embryonic fibroblasts. BMC Genomics 2008.

Series GSE11963

**References:**

1. Kupershmidt I, Su QJ, Grewal A, et al: Ontology-based meta-analysis of global collections of high-throughput public data. PLoS One 5, 2010

**Supplementary Figure Legends**

**Supplementary Figure 1. VLX1570-induced apoptosis in RPCI-WM1 cells.**

Representative heat density plot showing apoptotic cell death in RPCI-WM1 cells after treatment with DMSO or VLX1570 for 12 hr.

**Supplementary Figure 2. VLX1570 and b-AP15-induced apoptosis in primary WM cells.**

Representative heat density plots showing apoptotic cell death in CD19+/CD138+ tumor cells from WM patient cells (WM1 and WM2) after treatment with DMSO, VLX1570 or b-AP15 for 12 hr.

**Supplementary Figure 3. Mitochondrial membrane permeability is altered by VLX1570.**

Representative histograms are shown for BCWM.1/BR and RPCI-WM1 cells, where the black line represents isotype control and the red line indicates shift in TMRM fluorescence after VLX1570 (12hr) treatment. Statistical significance and percentage MOMP induced by VLX1570 in WM cells is presented in Supplementary Table 4.
