# Supplementary material for: Coinhibition of the deubiquitinating enzymes, USP14 and UCHL5, with VLX1570 is lethal to ibrutinib- or bortezomib-resistant Waldenstrom macroglobulinemia tumor cells
Source: Blood Cancer J. 2016 Nov 4;6(11):e492–. doi: 10.1038/bcj.2016.93 (PMC5148058; doi:10.1038/bcj.2016.93)
Supplement: Supplementary Materials and Methods [file bcj201693x5.docx]

**Supplementary Materials and Methods**

**Cell lines and authentication methods**

The BCWM.1 cell line was established by and obtained from Dr. Steven Treon (Dana Farber). The MWCL-1 cell line was established by and obtained from Dr. Stephen Ansell (Mayo Clinic Rochester). The RPCI-WM1 cell line was developed in our lab. Drug resistant clones from the 3 aforementioned cell lines were established in our lab. All cell lines were negative for mycoplasma contamination as tested on 8/4/16. These cell lines have not been previously STR profiled but will be submitted to Genetica DNA Laboratories - a LabCorp brand

**Immunoblot analysis**

Protein analysis using western botting techniques were carried out according to methods previously utilized by us.(1) Total protein extracts were prepared using RIPA lysis buffer with 0.2% protease and phosphatase inhibitor cocktail (Sigma, MO). Following centrifugation, the supernatant was collected for western blot analyses, and the protein content was measured by BCA protein assay reagent. 20µg of total protein were then boiled in Laemmli sample buffer, loaded onto 10% SDS-PAGE gels and transferred onto a nitrocellulose membrane. Blots were developed using chemiluminescence (Thermo Scientific, IL). Antibodies used are as follows:

CD184/CXCR4, BioLegend, Cat. # 306510

USP14, Santa Cruz, Cat. # sc-100630

UCHL5, Santa Cruz, Cat. # sc-271002

GAPDH, Santa Cruz, Cat.# sc-25778

BTK, Santa Cruz, Cat.# sc-1696

MYD88, Santa Cruz, Cat.# sc-74532

p-NFkB, Cell Signaling Technologies, Cat.# 3036S

NFATC2, Santa Cruz, Cat.# sc-7296

IRE-1a, Cell Signaling Technologies, Cat.# 3294

XBP-1s, Cell Signaling Technologies, Cat.# 12782

Caspase 9- Cell Signaling Technologies, Cat.# 9502

**Immunohistochemistry analysis on human tissues**

The Mayo Clinic Florida BAP/PRC Core Facility performed immunostaining on bone marrow aspirates collected from WM patients. Primary tissues were stained using USP14 and UCHL5. Slides were cut at 5um and air-dried overnight, deparaffinized and hydrated as usual. Antigen retrieval achieved using a DAKO Target Retrieval 6.0 pH solution with a steamer for 20 minutes at 90°C then allowed to cool for 20 minutes, rinsed in tap water and placed into DAKO TBS solution (Wash Buffer). Slides placed in 3% hydrogen peroxide (endogenous block) for 5 minutes then placed into DAKO TBS solution (Wash buffer). Primary antibodies were prepared using DAKO Antibody Diluent with Background Reducing Components: UCHL5 (Novus Biologicals – NBP1-85656 1:400 @ 1 hour RT), USP14 (Novus Biologicals – NBP1-86946 1:400 @ 1 hour RT); protein blocked for 5 minutes (DAKO – Serum Free) and stained using a DAKO immunostainer plus instrument (DAKO Cytomation, USA) using DAKO – Labelled Polymer HRP kits (Mouse/Rabbit EnVision+ System -30 minutes). Positive and negative controls used with each batch of slides stained, dehydrated/cleared as usual and counterstained with Gill I hematoxylin.

**Human Waldenström macroglobulinemia xenograft model**

All animal experiments were performed as previously described,(2) with the approval of the Institutional Animal Care and Use Committee of Mayo Clinic. Animals were monitored daily by animal care personnel. We calculated that with a sample size of 7 per group, 80% power at the 5% significance level to detect a difference in means of 1800 mm^3^ between the 2 groups could be achieved. For percentage change in IgM from baseline, with a sample size of 7 per group, we calculated 80% power at the 5% significance level to detect a difference in means of 450% between the 2 groups. Fourteen female NOD/SCID mice (6 – 8 weeks of age, purchased from Charles River) were subcutaneously implanted with 1x10^6^ luciferase labeled RPCI-WM1 cells (Luc-RPCI-WM1), which were allowed to grow till a bioluminescent signal was observed by IVIS imaging (Day 20). On day 21, mice were randomized into 2 groups (n=7 each), with one group receiving vehicle (cremaphor+PEG+Tween) and the other receiving VLX1570 at 4.4mg/kg via intraperitoneal injection. The investigator was not blinded to the group allocation. Both groups were respectively treated with either vehicle or VLX1570 every alternate day for 22 days. Sizes of the tumors was measured every 3 – 4 days using direct caliper measurements, and volume of the tumors was calculated using the formula (width)^2^ x length/2. Bioluminescent tumor imaging was performed with the Xenogen (IVIS) imaging system on Days 0, 20, 30, 36 and 43 post-tumor implantation. Blood from mice was collected on the same days by submandibular venous puncture, with sera subsequently separated for quantification of human IgM levels’ using ELISA. On Day 44, mice were sacrificed, and final tumor volume was measured in control and treatment arms. All images were obtained using a Canon D40 digital camera. No specific criteria for inclusion/exclusion were used as all mice formed tumors and were therefore included into the study.

**Immunohistochemistry analysis on mice tumor tissues**

Immunohistochemistry was performed on tumor sections from Luc-RPCI-WM1 xenografted mice treated with vehicle or VLX1570. Tissues were stained with IgM (Invitrogen 1:400), CXCR4 (Sigma-Aldrich 1:400), Ki-67 (DAKO 1:100), Ub-k48 (Sigma-Aldrich 1:100), or cleaved-caspase 3 (Cell Signaling 1:100) antibodies. Formalin fixed, paraffin embedded (FFPE) blocks were cut at 5 microns on positively charged slides and placed into a 60^o^C degree oven for 1 hour to dry. Slides were placed into xylene three times for 5 minutes each to remove paraffin. Slides were placed into decreasing grades of alcohol for 15 dips each (100% and 95% ETOH) to rehydrate tissues sections and were then placed into tap water for 5 minutes. Slides were placed into PBS buffer with Tween 20 for 5 minutes, and then were placed into 3% Hydrogen Peroxide for 5 minutes to block endogenous peroxidase. Slides were placed into PBS buffer with Tween 20 until IHC staining was started. Slides were placed onto a DAKO Immunostainer Plus and subsequently were scanned using the Leica Biosystems Aperio Digital Image Scanner XT (Leica Microsystems Inc. Buffalo Grove, IL USA). Positive Pixel Count from Aperio was used to determine intensity of antibody staining.

**Seahorse Cell Respirometry**

Mitochondrial oxygen consumption rate (OCR) was measured using the Seahorse XF96 flux analyzer (Seahorse Bioscience, MA, USA). BCWM.1 cells were treated with increasing concentration of VLX1570 in range 0 (DMSO) to 500 nM for two hours in cultivation medium. Cells were collected by centrifugation (RT, 10 min, 200xg) washed twice with custom made no phenol red containing low-buffered non-bicarbonated Seahorse medium pH 7.35: powder Dulbecco’s Modified Eagle’s Media supplemented with 10 mM glucose, 10 mM pyruvate and 2 mM L-glutamine (all from Sigma). Cell counts were adjusted and 2.5x10^5^ vital cells/well were seeded on Seahorse 96 well plate previously coated with poly-D-lysine – in pentaplicates per each condition. Pelleted cells (RT, 1 min, 600xg) were further caped in CO_2_-free incubator for 1 hour prior to the experiment. The Seahorse program included a 20 min probe equilibration step followed by a 3 min mix, and 3 min measurement, which was repeated three times before and after each injection. Drugs were injected sequentially in final concentration as follows: 2 μM Oligomycin, 1.5 μM FCCP and combination of Rotenone and Antimycin A each 0.5 μM (all from Sigma). After completion of the assay, obtained OCR values were normalized to total protein content. Cells in each well were lysed in RIPA buffer: 50 mM Tris-Cl pH 8.0, 150 mM NaCl, 0.1 % SDS, 0.5 % Deoxycholate and 1 % NP-40 (all from Sigma) and protein content was determined using BCA protein assay (Thermo Scientific) according to the manufacturer’s recommendation. Normalized data were analyzed by Seahorse Wave software and Excel macro file XF Mito Stress Test Report Generator. Displayed data show mean values and standard error of the mean.

**Computational modeling**

In silico drug-target interaction analysis was carried out as previously described.(3-7) Docking for VLX1570 and b-AP15 with USP14 (PDB: 2AYO) and UCHL5 (PDB: 3IHR), independently, was performed using Glide (v. 5.6) within the Schrödinger software suite (Schrödinger, LLC).(8)

**References**

1. Paulus A, Chitta K, Akhtar S, Personett D, Miller KC, Thompson KJ, et al. AT-101 downregulates BCL2 and MCL1 and potentiates the cytotoxic effects of lenalidomide and dexamethasone in preclinical models of multiple myeloma and Waldenstrom macroglobulinaemia. British journal of haematology. 2014;164(3):352-65.

2. Chitta K, Paulus A, Caulfield TR, Akhtar S, Blake MK, Ailawadhi S, et al. Nimbolide targets BCL2 and induces apoptosis in preclinical models of Waldenstroms macroglobulinemia. Blood cancer journal. 2014;4:e260.

3. Caulfield T, Devkota B. Motion of transfer RNA from the A/T state into the A-site using docking and simulations. Proteins. 2012;80(11):2489-500.

4. Caulfield T, Medina-Franco JL. Molecular dynamics simulations of human DNA methyltransferase 3B with selective inhibitor nanaomycin A. J Struct Biol. 2011;176(2):185-91.

5. Chitta K, Paulus A, Akhtar S, Blake MK, Caulfield TR, Novak AJ, et al. Targeted inhibition of the deubiquitinating enzymes, USP14 and UCHL5, induces proteotoxic stress and apoptosis in Waldenstrom macroglobulinaemia tumour cells. British journal of haematology. 2015;169(3):377-90.

6. Chou TC, Talalay P. Quantitative analysis of dose-effect relationships: the combined effects of multiple drugs or enzyme inhibitors. Advances in enzyme regulation. 1984;22:27-55.

7. Friesner RA, Murphy RB, Repasky MP, Frye LL, Greenwood JR, Halgren TA, et al. Extra precision glide: docking and scoring incorporating a model of hydrophobic enclosure for protein-ligand complexes. Journal of medicinal chemistry. 2006;49(21):6177-96.

8. Mohamadi F, Richard NGJ, Guida WC, Liskamp R, Lipton M, Caufield C, et al. Macromodel—an integrated software system for modeling organic and bioorganic molecules using molecular mechanics. J Comput Chem. 1990;11(4):440-67.
